# Characterization of latently infected EBV+ antibody-secreting B cells isolated from ovarian tumors and malignant ascites

**DOI:** 10.3389/fimmu.2024.1379175

**Published:** 2024-07-17

**Authors:** Lixin Zhang, Mary Strange, Esther Elishaev, Syed Zaidi, Francesmary Modugno, Mackenzy Radolec, Robert P. Edwards, Olivera J. Finn, Anda M. Vlad

**Affiliations:** ^1^ Department of Obstetrics, Gynecology and Reproductive Sciences, University of Pittsburgh School of Medicine, Pittsburgh, PA, United States; ^2^ Magee-Womens Research Institute, Pittsburgh, PA, United States; ^3^ Department of Pathology, University of Pittsburgh School of Medicine, Pittsburgh, PA, United States; ^4^ Magee-Womens Hospital of University of Pittsburgh Medical Center (UPMC), Pittsburgh, PA, United States; ^5^ Department of Immunology, University of Pittsburgh School of Medicine, Pittsburgh, PA, United States

**Keywords:** tumor infiltrating B cells, ovarian cancer, Epstein-Barr virus (EBV), B lymphoblastoid cell lines, antibody-secreting cells, CCDC155, GRB2, PDP2

## Abstract

**Introduction:**

Intra-tumoral B cells mediate a plethora of immune effector mechanisms with key roles in anti-tumor immunity and serve as positive prognostic indicators in a variety of solid tumor types, including epithelial ovarian cancer (EOC). Several aspects of intra-tumoral B cells remain unclear, such as their state of activation, antigenic repertoires, and capacity to mature into plasma cells.

**Methods:**

B lymphocytes were isolated from primary EOC tissue and malignant ascites and were maintained in cell culture medium. The stably maintained cell lines were profiled with flow cytometry and B cell receptor sequencing. Secreted antibodies were tested with a human proteome array comprising more than 21,000 proteins, followed by ELISA for validation. Originating tumor samples were used for spatial profiling with chip cytometry.

**Results:**

Antibody-secreting B lymphocytes were isolated from the ovarian tumor microenvironment (TME) of four different EOC patients. The highly clonal cell populations underwent spontaneous immortalization *in vitro*, were stably maintained in an antibody-secreting state, and showed presence of Epstein-Barr viral (EBV) proteins. All originating tumors had high frequency of tumor-infiltrating B cells, present as lymphoid aggregates, or tertiary lymphoid structures. The antigens recognized by three of the four cell lines are coil-coil domain containing protein 155 (CCDC155), growth factor receptor-bound protein 2 (GRB2), and pyruvate dehydrogenase phosphatase2 (PDP2), respectively. Anti-CCDC155 circulating IgG antibodies were detected in 9 of 20 (45%) of EOC patients’ sera. Tissue analyses with multiparameter chip cytometry shows that the antibodies secreted by these novel human B cell lines engage their cognate antigens on tumor cells.

**Discussion:**

These studies demonstrate that within the tumor-infiltrating lymphocyte population in EOC resides a low frequency population of antibody-secreting B cells that have been naturally exposed to EBV. Once stably maintained, these novel cell lines offer unique opportunities for future studies on intratumor B cell biology and new target antigen recognition, and for studies on EBV latency and/or viral reactivation in the TME of non-EBV related solid tumors such as the EOC.

## Introduction

Epithelial ovarian cancer (EOC), the most common type of ovarian cancer, is an aggressive disease, and the eighth leading cause of death among women worldwide (1). Diagnosis typically occurs late, when the lesions have spread beyond the ovaries, and into the peritoneal cavity. Histologically, EOC is highly heterogeneous, with the tumor microenvironment (TME) displaying a mixture of several cell types (epithelial tumor cells, innate and adaptive immune cells, stromal cells etc.), with different roles and prognostic values (2). A variety of immune cells can be found inside the tumor mass, including CD8+ tumor infiltrating T lymphocytes (TILs), which contribute positively to antitumor immunity and are correlates of improved prognosis (3–5). To enhance the number and cytolytic function of CD8 TILs, several immune therapeutic approaches have been tested to date, including immune checkpoint blockade, albeit with low clinical efficacy (6, 7). Advancing successful immunotherapies for EOC depends in part on the detailed profiling of the cellular composition of the immune TME and of the numerous tumor-immune cell interactions (8).

In addition to cytotoxic NK and CD8 TILs, tumor infiltrating B lymphocytes (B TILs) are also essential players in the anti-tumor response, and several recent studies have provided high resolution characterizations of the tumor ecosystem, at single cell level (9, 10). Although the antigen specificity and functionality of tumor-resident B cells have not been well characterized, the presence of plasma cells among the TILs seems to offer superior prognosis in EOC (11). Additionally, B TILs are often described in the context of intratumor tertiary lymphoid structures (TLS), and their coexistence with CD8+ TILs improves EOC clinical outcome (11–14).

Among the main attributes of B cells is the fact that they are highly efficient antigen presenting cells, especially when presenting low-abundance antigens to T cells (15, 16). They also play important roles in immune regulation, by secreting cytokines and chemokines (such as IFNγ, CXCL10, GM-CSF, IL-12, IL-7, CCL3-5) which can stimulate T cell chemoattraction, T and NK cell cytotoxic function and macrophage activity (10, 17, 18). In contrast, regulatory B cells, often in positive correlation with Tregs, have also been reported in various cancer types (19) and can exert immune inhibitory functions, often via IL-10 and IL-35 secretion (20, 21).

The overall fitness of memory T and B TILs depends on the inflammatory milieu and may also be influenced by earlier encounters that have impacted their genome. As the main target of the Epstein-Barr Virus (EBV, also known as the human herpes virus 4), human B cells can harbor this ubiquitous virus early and carry it in a latent phase throughout the lifetime of the host. More than 90% of the globe’s population (22) has been exposed to EBV, and B cells express CD21 and CD35, the major receptors for viral entry (23). Children exposed to EBV develop no or mild symptoms, whereas infection of adolescents can result in infectious mononucleosis (24). While EBV can infect both naïve and memory B cells, its prolonged persistence is through survival in a latent state in a subset of long-lived, memory B cells (25) that can evade immune surveillance. Since its first identification as an oncogenic virus that triggers transformation in Burkitt’s lymphoma (26), accumulating evidence points to EBV as the potential cause of several types of blood cancers and solid tumors (27, 28), and of autoimmune diseases such as multiple sclerosis and systemic lupus erythematosus (24).

Demonstrating EBV’s oncogenic potential, B lymphoblastoid cell lines have been previously established either spontaneously from the blood or lymphoid tissue of infected patients, or via *in vitro* EBV infection of blood circulating B cells (29). Collectively, these cell lines have been widely used for lymphomagenesis research models, or for mechanistic studies on the EBV-induced dynamic changes in B cell phenotypes (30). Similarly, *in vitro* exposure of tumor-isolated B cells to EBV has been used for the establishment of antibody-secreting lymphoblastoid cell lines (31–33), albeit with a low yield (32).

The quality and quantity of B TILs and their spatial distribution in the immune TME are important factors that influence tumor biology, and a better understanding of these factors may provide new opportunities for therapeutic interventions. Several aspects of intratumoral B cells remain unclear, such as their state of activation, antigenic repertoires, and capacity to mature into plasma cells. We report here that some of the tumor-resident memory B cells isolated from inflamed EOC cases (i.e. cases with high frequency of T and B TILs) are EBV positive, and that upon ex vivo culture, these EBV viral protein-expressing lymphoblastoid B cells become immortalized in an antibody-secreting state. Using protein arrays, we identified and validated the potential targets of the secreted antibodies. The newly described antigens are the coil-coil domain containing protein 155 (CCDC155), growth factor receptor-bound protein 2 (GRB2), and pyruvate dehydrogenase phosphatase2 (PDP2), respectively. The antibodies recognize their targets in the tumor microenvironment, raising new venues of exploration of their antigenic and immunogenic potential in EOC. These studies provide new insights on EBV status in B TILs and supply a platform for immortalization of tumor resident, naturally infected B cells, isolation and characterization of their secreted antibodies and antigen identification.

## Materials and methods

### Primary tumor tissue, ascites, PBMCs and ovarian cancer cell lines

All patient samples were obtained deidentified, according to approved institutional IRB protocol, and after patient consent. Sample processing occurred within 2-3 hours from collection time. Fresh tumor tissue was washed with DPBS (Corning), cut into 1 to 2 mm^3^ pieces and put into a T75 flask (Corning) with 15 ml complete RPMI-1640 (RPMI supplemented with 10% fetal bovine serum (FBS, Gibco), 10,000 U/L penicillin (Sigma), 10,000 U/L streptomycin (Sigma), and 2 mmol/L L-glutamine (Gibco). Cells were maintained at 37°C, 5% CO_2_, and fresh culture medium was added every 3-5 days. Cells were trypsinized at confluency, using TrypLE™ Express (Gibco), and the tissue organoids and 1/5 of the cells were replated. For B lymphoblastoid cell lines, after about six weeks, the lymphocyte-like cells were collected by pipetting and moving to a new flask and cultured with complete RPMI. For human ovarian cancer cell lines, attached stromal cells were removed by short period trypsinization (2-3 mins) while the epithelial tumor cells were still attached to the flask, followed by trypsinization for another 5 to 10 minutes to recover the tumor cells, 1/5 of which were replated. Cells were propagated for 15 to 20 passages to form cell lines.

Ascites fluid was centrifuged at 400 g, 10 minutes. Pelleted cells were placed in a T75 flask in 20 ml complete RPMI-1640 and cultured long term.

Peripheral blood mononuclear cells (PBMCs) from female healthy donors were purchased from STEMCELL technologies and cultured in the same conditions.

All EOC cell lines and the human EBV-related Burkitt lymphoma cell line Raji were obtained from ATCC, except for UP04, UP13 and UP14 cell lines, which were derived in house from primary tissue. All cancer cells were cultured in complete RPMI-1640.

### Antibody purification, isotype determination and PE/R-Phycoerythrin conjugation

The B cells derived from ovarian cancer (BOC cell lines) were transferred to serum-free AIM-V medium (Gibco) for a minimum of 5 days (and up to three weeks) prior to antibody purification, so only the BOC- secreted immunoglobulins would be pulled down by the protein A/G beads (34). Cell free culture supernatants were co-incubated with protein A/G Plus agarose (Pierce) or PureProteome™ Kappa Ig Binder Magnetic Beads (Millipore) at room temperature (RT) for 60 minutes. After three washes in PBS, antibodies were eluted with 0.2 M glycine-HCl, pH2.2 followed by neutralizing 1M Tris-HCl solution, pH8.9. A fraction of isolated IgG (5μg) was loaded onto 4-20% SDS-PAGE to check for purity. Purified antibodies were conjugated with PE (Ab36, Ab73) or PerCP-Cy5.5 (Ab89) using PE/R-Phycoerythrin or PerCP-Cy5.5 Conjugation Kit - Lightning-Link^®^ (Abcam). Isotypes of purified antibodies were determined using Pro-Detect Rapid Antibody Isotyping Assay Kit-Human (ThermoScientific) according to the provided protocol. Serum IgG fraction was purified using caprylic acid (35) and sodium sulfate precipitation, as previously described (36).

### Flow cytometry

Cells were stained with fluorescent labeled primary antibodies or isotype control in 1% bovine serum albumin (BSA, Fisher Bioreagents) in DPBS, on ice, for 30-40 min, and the data were acquired with LSR II and analyzed with FACSDiva or FlowJo (all from BD). The following anti-human antibodies were used: FITC-CD19 1:5, FITC-CD20 1:5, APC-CD27 1:5, BV421-anti-human IgD 1:100, PE-Cy7-CD28 1:100, PE-anti-human IgG 1:5 (all from BD), PE-CD138 1:100 and PerCP-Cy5.5-CD45 1:100 (both from BioLegend).

### Chip cytometry

Cells (1x10^6^) suspended in 100 μl DPBS were loaded onto cell chips (ZellSafe, Canopy Biosciences) and allowed to settle for 5-10 minutes at RT. The cell chips were washed with 1 ml DPBS, fixed with fixation buffer (Canopy Biosciences) for 45 minutes at 4°C, and washed with 1 ml DPBS followed by 1 ml ZELLKRAFTWERK storage buffer (Canopy Biosciences). Cells were stained with PE-anti-human Ig, kappa 1:100 BV421-anti-human Ig, lambda 1:100, PE-anti-human IgG1 1:100, BUV395-anti-human IgG3 1:100 (all from BD), PE-Ab36 1:100. All antibodies were titrated in 300 μl of ZELLKRAFTWERK storage buffer and incubated at RT for 5 minutes. After washing the cell chips with 5 ml DPBS two times at a 2-minute interval, images were acquired with ZellScanner ONE or CellScape™ and analyzed with ZKWapp DataWizard (Canopy Biosciences). Tissue chips were mounted with 4 μm sections from formalin fixed and paraffin embedded (FFPE) tissue blocks following the protocol provided by Canopy Biosciences and stained with AF488-anti-CA125 1:100 (R&D systems), PE-anti-Vimentin 1:500 (BD), PerCP-Anti-CD3 1:100, PerCP-anti-EpCAM 1:100 (Canopy Biosciences Spatial Immune Profiling Kit), PE-Ab36 1:100, PE-Ab73 1:100 and PerCP-Cy5.5-Ab89 1:300 (labeled in house with PE, or PerCP-Cy5.5 according to manufacturer’s instructions and as described above), at room temperature, for one hour.

### Immunohistochemistry

Sectioning of formalin-fixed, paraffin embedded tumor blocks into four-micron sections, and hematoxylin/eosin (H&E) staining were performed at the Magee-Womens Research Institute Pathology Core. Histopathology examination was performed by a trained pathologist (EE). Blank slides were used in house for IHC. Slides were left at 56°C overnight and rehydrated with xylene, 100% ethanol, 95% ethanol, 70% ethanol and water. Peroxidase blocking with 3% H_2_O_2_/methanol (Fisher Chemical) and antigen retrieval was performed by boiling the slides for 20 minutes in pH 9 Tris-EDTA buffer. The slides were blocked with 1% BSA in DPBS, 30 min at RT. The positive signal was detected using the ImmPACT DAB EqV Substrate Kit (Vector Laboratories, Burlingame, CA) and the slides were counterstained with hematoxylin. To ensure specificity of staining, control sections were stained with HRP-conjugated human IgG isotype control.

Single stain for PNAd (BioLegend, clone MECA-79, 1:50) was performed using Tris-EDTA (pH 9, 20 min). Biotin mouse anti-rat IgM Antibody (BioLegend, clone MRM-47, 1:100) was used as secondary antibody, followed by Pierce™ High Sensitivity Streptavidin-HRP (Thermo Scientific, 1:500). Agilent Liquid DAB+ Substrate Chromogen System was used as chromogen. For CD3/CD20 dual IHC, the protocol provided with Vector ImmPRESS Duet Double Staining Polymer Kit was used to carry out the staining. Mouse anti-human CD3 (Agilent, Clone F7.2.38, 1:100) and rabbit anti-human CD20 (Abcam, Clone EP459Y, 1:200) were used as the primary antibodies. Images were captured with Axiocam 105 color camera on ZEISS Light Microscope.

### Western blot

Cell pellets were washed with cold DPBS and lysed with RIPA (Pierce, Rockford, IL, USA) supplemented with Halt protease inhibitor cocktail, Halt phosphatase inhibitor cocktail and 0.05 M ethylenediaminetetraacetic acid (Thermo Scientific, Rockford, IL, USA). Protein lysates (10–20 μg) were loaded onto 4–20% Mini-Protean precast gel (Bio-Rad Laboratories) and run at 150V for 1h and transferred at 120 mA for 90 minutes to nitro-cellulose membranes (Bio-Rad Laboratories). Membranes were incubated in blocking buffer -2% BSA (Fisher Scientific), at RT for 1h and then blotted with primary antibodies in blocking buffer with 0.05% Tween-20 (Fisher Bioreagents), at 4°C overnight. Primary antibodies to the following antigens were used: EBV latent membrane protein 1 (Abcam, [CS 1-4], ab78113, 1:2000), β-actin (Sigma, A1978, 1:5000). CCDC155 (polyclonal antibody (Sigma-Aldrich, HPA019940, 200 mg/ml, 1:1000), human Ig kappa light chain (R&D Systems, Cat. # MAB10050-100, 1:2000), human Ig, lambda light chain (clone 1000040, R&D Systems™, 1:2000), mouse-anti-GST (Abcam, ab92, 3G10/1B3, 1:5000).

Goat-anti-mouse (170–6516) or goat-anti-rabbit (170-6515) IgG-HRP (both from Bio-Rad), or rabbit-anti-human IgG-HRP (Dako, 1.34 g/L) were diluted 1:6000 in blocking buffer. SuperSignal West Femto kit (Thermo Scientific) was used to develop the membranes. Images were taken with Chemidoc XRS darkroom system (Bio-Rad).

### PCR

Primers (IDT) used for all PCR protocols are listed in **Table 1**. Two EBV genes - *LMP1* (37) and *EBNA3C* (38)- were detected using two sets of primers, respectively. Wild type *LMP1* shows up as a band of 316 bp, while *LMP1-Del30* has a band of 286 bp. Type I virus shows a *EBV3C* band of 153 bp and type II of 246 bp. PCR conditions: 95°C, 3 min; (95°C, 30s; 58°C, 30s; 72°C, 1 min) x 40; 72°C, 5 min; 12°C hold. 1.5% TAE gel, 120v, 40 min.

**Table 1:** PCR primers.

Name	Sequence*
*LMP1*-Forward	5′‐AGCGACTCTGCTGGAAATGAT‐3′
*LMP1*-Reverse	5′‐TGATTAGCTAAGGCATTCCCA‐3′
*EBV3C*-Forward	5’-AGAAGGGGAGCGTGTGTTGT-3’
EBV3C-Reverse	5’-GGCTCGTTTTTGACGTCGGC-3’
*CCDC155* Forward	5’-CAGCCTAGCCAGTGACATCC-3’
*CCDC155* Reverse	5’-TGGGGACTCACCCATGTAGT-3’
*CCDC155* F1	5’-GTTCCG *GGATCC* ATGGACCTGCCCGAG-3’
*CCDC155* R1	5’-CACGAT *GCGGCCG C* CAGAGCCAAGATCTC-3’
*CCDC155* F2	5’-GTTCCG *GGATCC* TTGCAGCGGAGCATG-3’
*CCDC155* R2	5’-CACGAT *GCGGCCGC* TTTCTGTCGAATGGC-3’
*CCDC155* F3	5’-GTTCCG *GGATCC* CAGCTGAGAAGAGTG-3’
*CCDC155* R3	5’-CACGAT *GCGGCCGC* CTCTCACACTGGAGG-3’
*CCDC155* F4	5’-GTTCCG *GGATCC* GAGATCTTGGCTCTG-3’
*CCDC155* R4	5’-CACGAT *GCGGCCG C* CAGATGCTGCTGCTC-3’
*CCDC155* F5	5’-GTTCCG *GGATCC* GAACAGAATCGCAGC-3’
*CCDC155* R5	5’-CACGAT *GCGGCCGC* AGTGCGCTCAGAGAG-3’
*CCDC155* F6	5’-GTTCCG *GGAT CC* TTGAAGCGGCAGCTC-3’
*CCDC155* R6	5’-CACGAT *GCGGCCGC* TCTTCTCAGCTGGGC-3’

*Italicized sequences mark the BamHI and NotI restriction enzyme cut sites.

PCR conditions for *CCDC155*: ~90ng DNA templates; 94°C 3min; (94°C, 30s;58°C,30s;72°C, 1min) x40; 72°C, 5min; 12°C. 1.0% TAE gel, 120v, 40 min. Wild type gene appears as a single band. Upon knock-out, multiple bands or smears will appear due to the DNA repair attempts.

A total of six pair of primers (**Table 1**) were used for antibody epitope mapping. BamHI and NotI restriction enzymes cut sites (**Table 1**, *italic*) were inserted into forward and reverse primers, respectively, for cloning purposes.

Templates for *CCDC155* F1/R1, F2/R2 and F3/R3: 1ng pcDNA3.1-CCDC155 (GeneScript, OHu32184), 94°C 3min; (94°C, 30s;65°C,30s;72°C, 1min) x35; 72°C, 5min; 12°C. 1.0% TAE gel, 120v, 33 min. 10 μl loaded.

Templates for *CCDC155* F4/R4 and F5/R5: 10ng pcDNA3.1-CCDC155 in 200μl total reaction volume. 94°C 3min; (94°C, 30s;67°C,30s;72°C, 1min) x 37; 72°C, 5min; 12°C. 1.5% TAE gel, 120v, 32min, 8 μl loaded.

### BCR sequencing

Genomic DNA was extracted using Qiagen AllPrep DNA/RNA Micro Kit and sent to Adaptive Biotechnologies for BCR sequencing. Sequencing data were analyzed using immunoSEQ Analyzer 3.0.

### Proteomics array for target identification

Purified antibodies were shipped to CDI LABS, to be analyzed with the High-Spec^®^ cross-reactivity assay on the HuProt™ human proteome array, which contains >21,000 unique, full‐length, individually purified human proteins on a single microscope slide. According to the manufacturer’s description, the full‐length recombinant proteins are expressed in the yeast S. cerevisiae, purified, and printed on glass slides in duplicate, along with a set of control proteins (GST, BSA, histones, IgG, etc.). The HuProt™ expression library was created by inserting full-length human open reading frames (ORFs) into a yeast high-copy expression vector that produces GST-His6 fusion proteins when induced in yeast (39, 40). The company-provided protocol describes that the arrays were incubated with blocking buffer (1xTBS/0.1% Tween 20/5% BSA) at room temperature for 1 hr, with gentle shaking. Antibodies were diluted to 1 μg/mL in blocking buffer and probed on the arrays for 1 hour. After probing, the arrays were washed three times with TBST (1xTBS/0.1% Tween 20) for 10 min, and then exposed to Alexa647-anti-mouse IgG Fc secondary antibody (0.25 μg/mL) for 1 hour in a light-proof box with gentle shaking, followed by 3 washes with TBST for 10 min each and 3 rinses with ddH_2_O. The arrays were then dried with an air duster and scanned using a GenePix 4000B scanner for data collection. Non-specific hits (due to binding to the secondary antibody) were eliminated from the analysis. CDI software was used to quantify binding to specific proteins on the array, based on Z Scores, as described in the statistical analysis section.

### Ab36 epitope mapping

Six pair of primers were designed to amplify CCDC155 cDNA fragments (further detailed in the “PCR” section). The full CCDC155 protein contains 562 amino acids (aa). The first three pairs of primers were used to amplify fragments F1, F2, and F3, encoding aa 1 to 204, aa 182 to 383, and aa 358-562, respectively, with a 22 aa overlap between F1 and F2, 25 aa overlap between F2 and F3. The amplified fragments were digested with BamHI and Not I (Thermo Fisher), at RT for 10 minutes, and linked into BamHI and Not I digested pGEX-4T-1 (Cytiva, Product No. 28954549) vector, respectively, using the Rapid DNA Ligation Kit (Thermo Fisher). The constructed vectors (5 μl) were transformed into 50 μl BL21 (DE3) competent cells (Thermo Fisher) at 42°C for 30 seconds, followed by incubation on ice, for 2 minutes. After adding 250 μl of S.O.C. Medium (Thermo Fisher), the transformed *E. coli* BL21 (DE3) cells were placed on a shaker, at 200 rpm, 37°C for 1 hour. The *E. coli* cells from each transformation reaction were spread on separate LB plates containing 100 μg/ml ampicillin (Thermo Fisher) and incubated overnight at 37°C. Colony PCR was performed using the same primers and conditions for amplification of the corresponding fragments, as described in the PCR section. Three positive colonies from each transformation were picked, placed in 3 ml LB medium with 100 ug/ml ampicillin at 200 rpm, 37°C overnight and 1:100 diluted in 3 ml fresh LB medium with 100 ug/ml ampicillin and 0.5M IPTG (Thermo Fisher), 200 rpm, 37°C for 4 hours. Cell pellets from 1.5 ml were resuspended in 500 ul 1x SDS-PAGE loading buffer (Bio-Rad), ultrasonic treatment (Sonics GEX130 Ultrasonic Processor) for 30 seconds and heated at 95°C for 5 minutes. SDS-PAGE and Western blot were used to confirm the expression of the protein fragments.

### ELISA

Immulon 4 HBX ELISA plates (Thermo Fisher) were coated with one of the following proteins: CCDC155-GST, CPNE1-GST, PDP2-GST, NO66-GST, GMPS-GST, PTPN2-GST (all from CDI). Recombinant S. japonicum GST protein (Abcam) was used as control. All proteins were diluted in DPBS at 4 μg/ml, 100 μl/well in duplicates, incubated at 4°C overnight. Excess protein was washed four times with PBS-T (with 0.05% Tween-20) and blocking buffer (1% BSA in PBS) was added for one hour at RT. Purified antibody was added at 2 μg/ml, 100 μl/well. Human serum/plasma were 1:50 diluted with blocking buffer and added at 100 μl/well. Both were incubated for one hour at RT. HRP-rabbit-anti-human IgG, 1:5000 (Dako), HRP-mouse anti-human IgG3 (hinge) 1:1000, HRP-goat anti-human IgG1 (heavy chain) 1:1000, HRP- goat anti-human lambda light chain (1:2000), HRP- goat anti-human kappa light chain (1:2000), (all four from Invitrogen), or AP-goat-anti-human IgG (1:5000, Sigma) were added at 100 μl/well for one hour at RT. Reaction was developed using TMB (BD) at RT for 12 to 15 minutes or p-Nitrophenyl phosphate (Sigma) at RT for 20 minutes, and plates were read at 450 nm or 405 nm, respectively with Infinite 200 Pro ELISA plate reader (Tecan). The reported values represent optical density units at 450nm (OD450) and were calculated after subtraction of the anti-GST background, as follows: CCDC155 (OD450) = CCDC155-GST (OD450) - GST (OD450). The same formula was applied to samples read at 405nm wavelength.

### CRISPR-mediated deletion of *CCDC155*

Ovarian cancer TOV21G cells (4 x 10^5^ cells in 3 ml cRPMI/well) were seeded in 6-well tissue culture plate, without antibiotics. UltraCruz™ Transfection Reagent (Santa Cruz Biotechnology, sc-395739, 10 µl) was diluted in 140 µl Plasmid Transfection Medium (Santa Cruz Biotechnology, sc-108062). CCDC155 double nickase plasmids (2 µg) (Santa Cruz Biotechnology, sc-410329-NIC) or control plasmids (2 µg) were added into Plasmid Transfection Medium (Santa Cruz Biotechnology) to final volume of 150 µl, mixed with the transfection solution and added dropwise to the well containing TOV21G cells in 2ml fresh antibiotic-free growth medium. After culturing for 24 hours, cells were trypsinized with 1 ml/well Gibco™ TrypLE™ Express, washed and analyzed via flow cytometry to check the transfection efficiency. GFP sorting and puromycin selection were used sequentially to achieve high yield (~90%) of *CCDC155* gene knockout. GFP positive cells were sorted, resuspended in 10 ml cRPMI without antibiotic and divided into four wells (3x10^4^/well/2.5ml) in a 12-well plate and cultured overnight, followed by adding puromycin to 400, 500, 600, 700 ng/ml cRPMI, respectively, for a 7-day selection. PCR and Western blot were used to confirm the loss of CCDC155.

### Transfection with plasmid encoding for human CCDC155 protein

OVCA432 cells were seeded in a well of a 6-well plate, 1x10^6^ cells/well in cRPMI and cultured overnight at 37°C, 5% CO_2_. Cells were transfected with either CCDC155_OHu32184D_pcDNA3.1+/C-(k)-DYK (3 µg) or control pcDNA3.1+ (3 µg) (GenScript) using UltraCruz^®^ Transfection Reagent (Santa Cruz Biotechnology). The transfected cells were cultured for 48 hours and CCDC155 expression was confirmed by Western blot.

### Statistics

Z Score is the average Z Score of the duplicate spots of a given protein (each protein was printed in duplicate on the HuProt™ array). The Z Score of each spot on a given array is calculated with the formula Z= [F635 – F635(avg)]/F635(std).

F635(avg) and F635(std) are the average and standard deviation of the F635 values of all spots on the array, respectively. F635 is the average foreground signal intensity of 2 replicate spots of a given protein in the detection channel (IgG = 635 nm). The complete statistical methodology has been previously reported (41).

## Results

### Patient characteristics

We used clinical specimens from four different EOC cases, with disease characteristics detailed in **Table 2**. All patients had high grade serous EOC, FIGO stage IIIc (3 patients) or IIIb (one patient). Two of the samples were chemo-naïve and were obtained at the time of primary surgical debulking, while the other two were collected at the time of interval surgery, 27 and 16 days post completion of neoadjuvant chemotherapy, respectively, according to standard of care.

**Table 2:** Patient demographics.

Cell line name	*BOC-36	BOC-73	BOC-78	BOC-89
**Cell line originating site**	Tumor	Tumor	Ascites	Tumor
**Patient ID**	19-36	21-73	22-78	22-89
**Tumor biopsy ID** **(Collection time)**	19-36 Tumor (Interval, 27 days**)	21-73 Tumor (Primary)	22-78 Tumor (Primary)	22-89 Tumor (Interval, 16 days)
**Age range at diagnosis**	70-75	75-80	70-75	65-70
**Histology**	High Grade Serous	High Grade Serous	High Grade Serous	High Grade Serous
**Stage**	IIIc	IIIc	IIIb	IIIc
**Chemo status**	Post-NACT***	Chemo naïve	Chemo naïve	Post-NACT

*BOC, B cells derived from ovarian cancer; **Days post-NACT ***NACT- neoadjuvant chemotherapy.

### Spontaneous immortalization of B lymphoblastoid cell lines after prolonged *in vitro* culture of primary cells isolated from ovarian tumor tissue and malignant ascites

**Figure 1:**
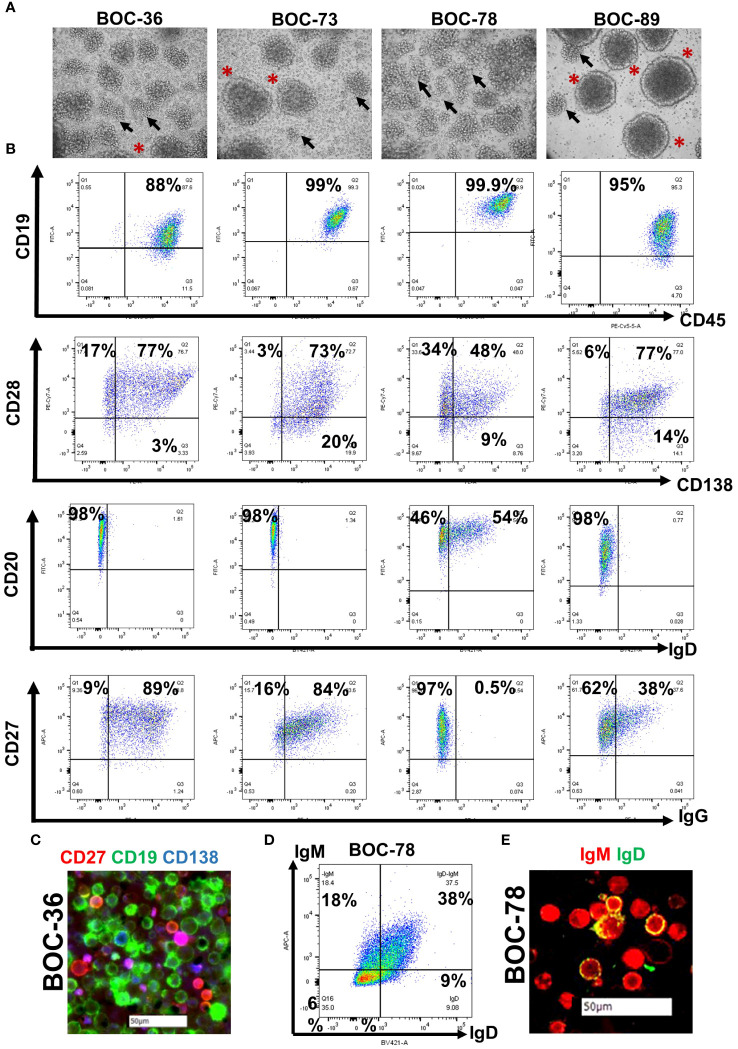
All four newly established cell lines show lymphoblastoid characteristics and are positive for B cell markers. **(A)** Visualization of stably cells in culture, cultured in T75 flasks, using a camera attached to inverted microscope (20 × magnification); Arrows- cell clumping; asterisks- rosette shape morphology. **(B)** Dot plot analysis of cell surface marker distribution (indicated on x and y axes), using multicolor flow cytometry. Gates were set using isotype controls. Percent positive cells are shown. **(C)** Morphological characterization of BOC-36 cells and assessment of marker distribution using chip cytometry. Scale bar (white)- 50μm. **(D, E)** IgM and IgD expression on BOC-78 cells using flow **(D)** and chip cytometry **(E)**. Scale bar (white)-50μm.

All samples (three fresh tumor tissue and one malignant ascites, **Table 2**) were handled as described in Materials and Methods, and as outlined in the study diagram (**Supplementary Figure 1**). Following tissue processing, all cell populations exhibited the expected morphological heterogeneity, with cancer cells, fibroblasts and immune cells being visible in the cell culture flask (**Supplementary Figure 2A**). After several weeks in culture, outgrowth of a single cell phenotype was observed. The rapid expansion indicated that cells have entered the stage of exponential proliferation, and the growth pattern showing cell clumping and rosette morphology (42) was suggestive of a proliferating lymphocytic population (**Figure 1A**). Cells were continuously propagated *in vitro* for an average of 15 weeks, until stably maintained. Multi-parameter flow cytometry and chip cytometry data (**Figures 1B, C**) revealed that the established cells are homogeneously positive for the pan-leukocyte marker CD45, negative for the CD3 T cell marker, and positive for CD19 and CD20, indicating that all four lines are lymphoblastoid B cells (**Figure 1B** and **Supplementary Figure 2B**), exhibiting the expected size, morphology, and cell surface marker distribution (**Figure 1C**). Importantly, all cells are positive for the CD27 memory marker (43) and express high levels of plasma cell markers CD138 (44) and CD28 (45). Three of the four cell populations (BOC-36, BOC-73, and BOC-89) are IgG positive (**Figure 1B**). The fourth cell line (BOC-78) is IgM positive, with most IgM+ cells co-expressing IgD, as shown by flow (**Figure 1D**) and chip cytometry (**Figure 1E**). Altogether, these phenotypic markers point to an antigen-experienced, IgG or IgM memory plasma cell phenotype.

### B cells originate in tumors with T and B cell conglomerates

**Figure 2:**
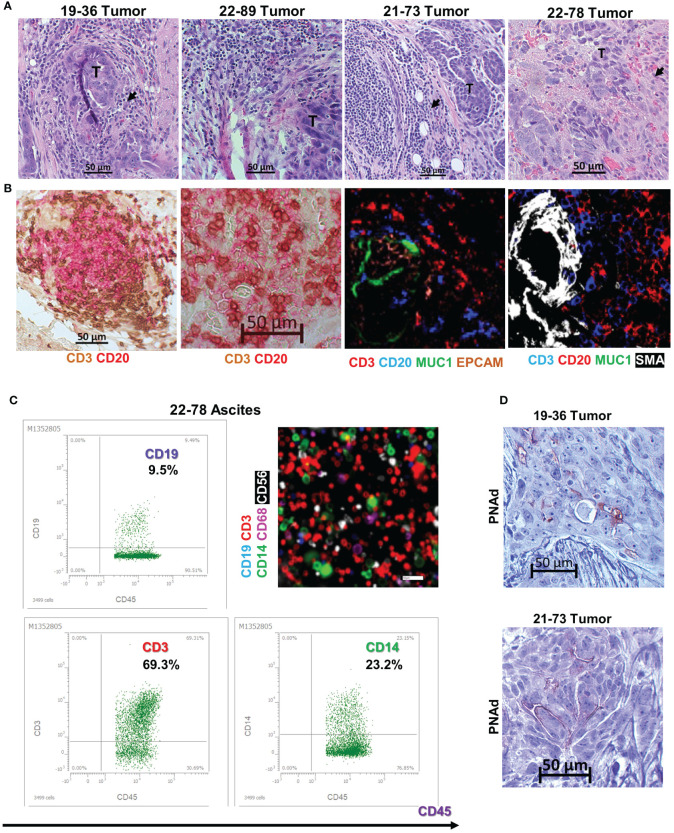
B cell lines originate in tumors with inflamed phenotypes. **(A)** Tissue histology using hematoxylin and eosin (HE) stain. Tumor areas (T) surrounded by small mononuclear cell infiltrates suggestive of TILs (arrowheads). Scale bars- 50μm. **(B)** T and B cell intratumor distribution using dual color immunohistochemistry (left two panels- CD3- brown; CD20- red) and chip cytometry (right two panels, colors as indicated), Scale bar- 50μm. **(C)** Immune phenotyping of ascites cells using flow cytometry (dot plots, upper left and lower left, and right panels). Cells were gated on CD45+ cells, percent double positive cells are shown. Gates were set using isotype controls for each marker. Chip cytometry images of ascites cells (upper right panel). Scale bar, 50 μm. **(D)** Immunohistochemistry staining for PNAd in tumor samples 19-36 (origin of BOC-36) and 21-73 (origin of BOC-73). Scale bar, 50μm.

Histopathologic examination of the originating tumor tissue reveals an immune inflammatory tumor profile, with presence of tumor infiltrating, small mononuclear cells visible on hematoxylin-eosin (HE) staining (**Figure 2A**). The cells are positive for T and B cell markers on dual stain IHC or tissue chip cytometry (**Figure 2B**). The T and B cell infiltrates resemble lymphoid aggregates (**Figure 2B**), the hallmark of inflamed tumor phenotypes (46). The starting material for cell line BOC-78 was ovarian cancer ascites fluid (sample 22-78-Ascites, **Table 2**), consisting of mostly CD45+ inflammatory immune cells, including CD3+ T and CD19+ B lymphocytes, CD14+ monocyte/macrophages and CD56 NK cells (**Figure 2C**). Importantly, this patient’s corresponding tumor tissue (sample 22-78-Tumor) also shows presence of infiltrating CD20+ B cells, mostly as T-B cell conglomerates (**Figure 2B**). Of the four samples, staining for peripheral lymph node addressin (PNAd), important for lymphocyte homing, was positive in tumor samples 19-36 (the source of BOC-36, **Table 2**) and 21-73 (the source of BOC-73, **Table 2**), suggesting possible TLS formation in these two cases (**Figure 2D**). For the other two PNAd negative cases (22-89 Tumor and 22-78 Tumor), we classify the T and B cell infiltrates as lymphoid aggregates. Together, these results point to the presence of a B TIL-rich environment and presence of intratumor T/B lymphoid conglomerates (either as aggregates or TLS) (11, 47) in all four EOC cases.

### Spontaneously immortalized lymphoblastoid B cells are EBV positive

**Figure 3:**
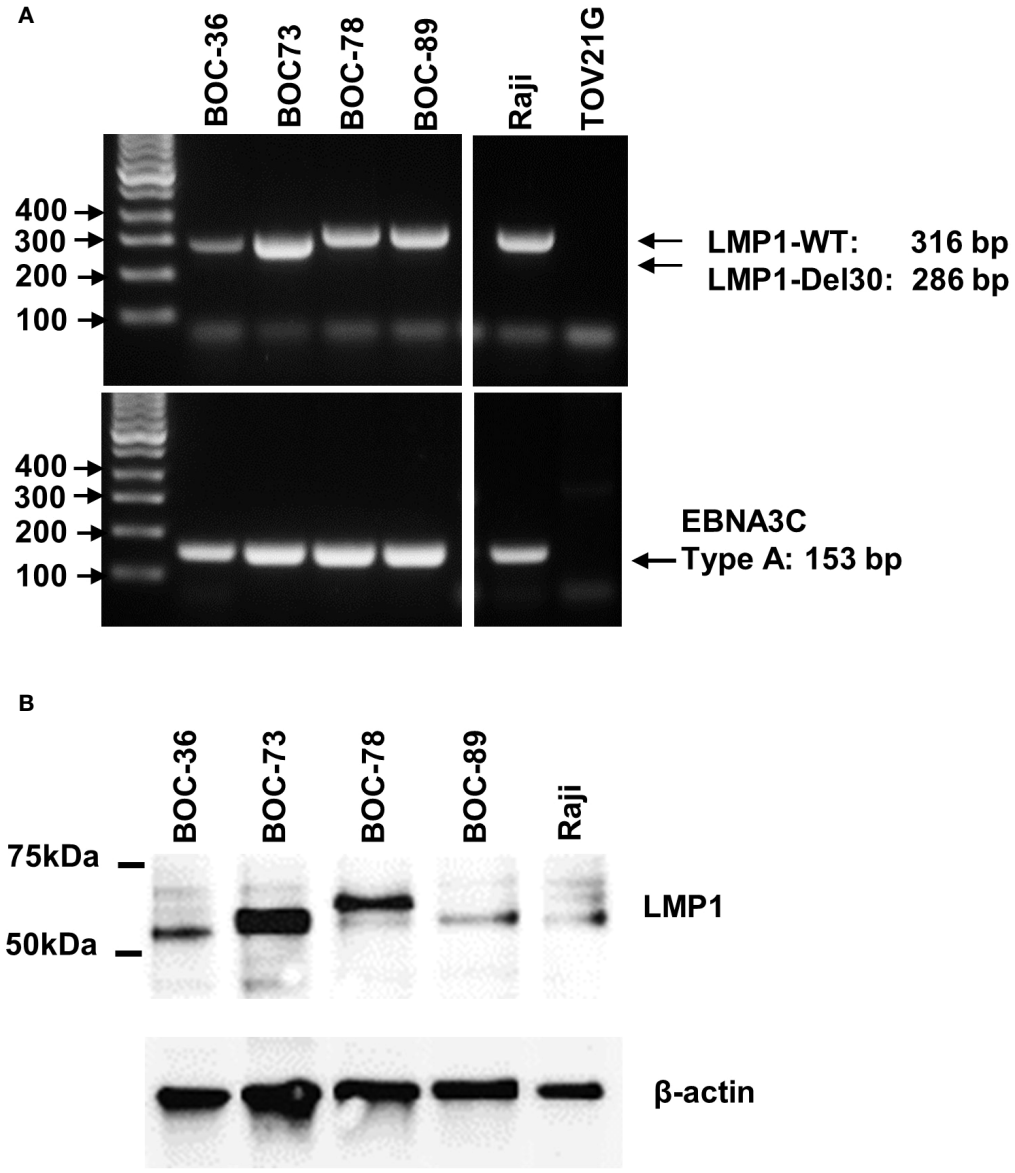
All four cell lines are EBV positive. **(A)** Detection of LMP1 (top) and EBNA3C (bottom) EBV viral genes by PCR, using DNA extracted from the four lymphoblastoid (BOC) cell lines, and the originating tumor or ascites. DNA from RAJI B lymphoma and TOV21G ovarian cancer cell line were used as positive and negative controls, respectively. Lane 1- DNA mass ladder. **(B)** Detection of EBV LMP1 viral protein by western blotting using cell lysates, as indicated. Raji cells were included as EBV positive control. Beta actin was used as loading control. Molecular weight size markers are shown on the left.

To identify the potential cause for the observed spontaneous immortalization, we interrogated all four B lines for the presence of EBV, an oncogenic virus with tropism for and latency in memory B cells. PCR analyses revealed the presence of LMP1 and EBV3C viral genes in all four cell lines (**Figure 3A**). The LMP1 PCR product from samples BOC-78 and BOC-89 had a size consistent with the wild-type sequence (316 base pairs, bp), also found in the well characterized, EBV positive RAJI B lymphoma cell line (48), included as positive control. The smaller size PCR products detected in BOC-36 and BOC-73 suggest that these patients had been infected with the LMP1 30-bp deletion variant (49). The presence of EBNA3C 153 bp product points to type-A EBV in all cell lines (**Figure 3A**) (49). Western blot results revealed viral LMP1 protein expression in all four cell lines (**Figure 3B**), confirming viral activity. The observed size difference may be caused by genetic variations in *LMP1*, such as deletions and/or different numbers of tandem repeats, as previously described (50).

### Spontaneously immortalized lymphoblastoid B cells are highly clonal and secrete antibodies of different isotypes

**Figure 4:**
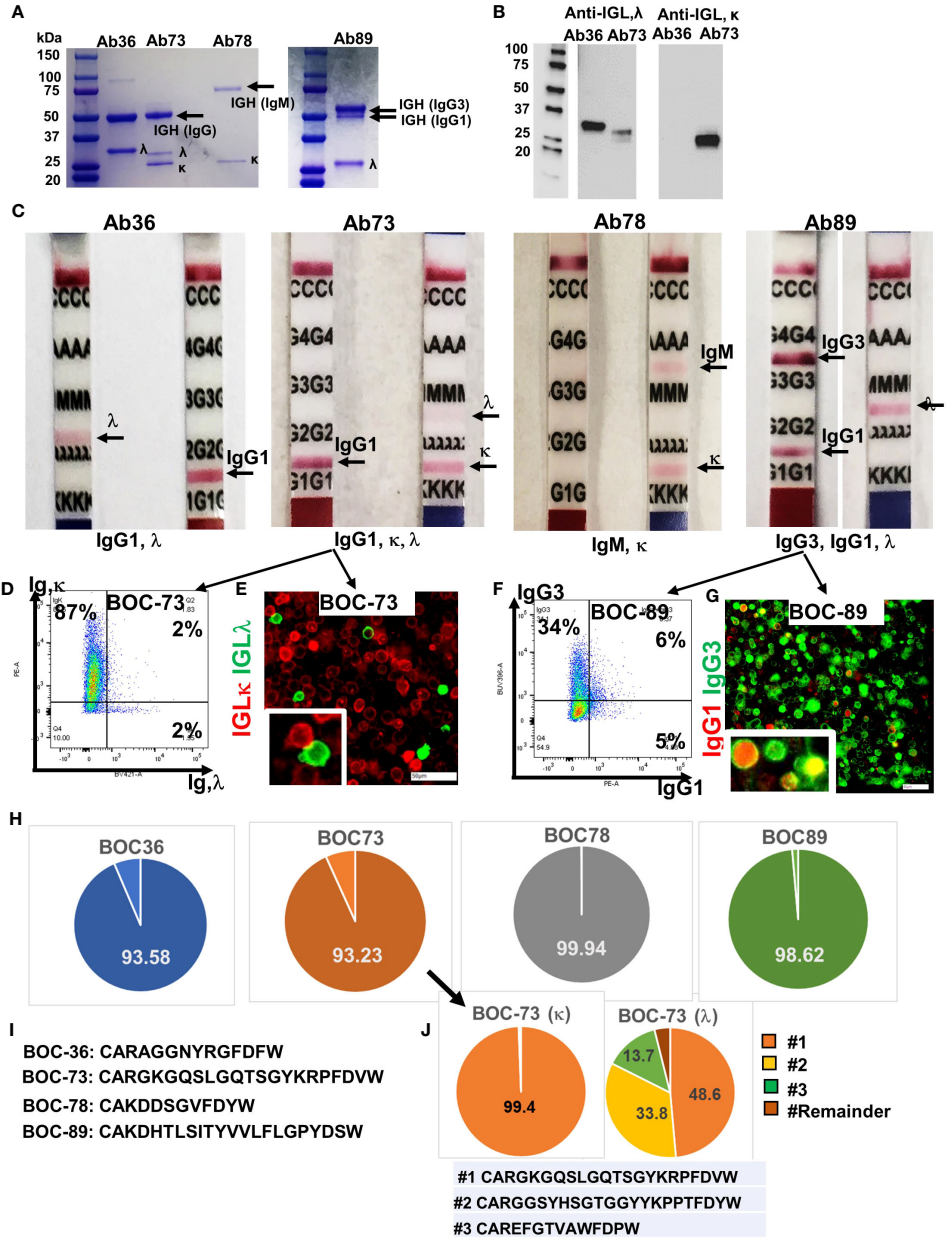
All four cell lines secrete immunoglobulins. **(A)** SDS- PAGE (4-20%) detection of purified immunoglobulins (heavy and light chains), secreted in cell culture medium by the four BOC lines. First lane, molecular weight ladder. **(B)** Western blot of Ab36 and Ab73, using detection antibodies specific to either IGL,λ (left) or IGL,κ (right). Left panel, molecular weight panel. **(C)** Purified antibodies from each cell line were loaded onto Ig isotype detection strips. Positive results are indicated by a red line. **(D)** Flow cytometry of BOC-73 cells stained for IGL, κ (y axis) and λ (x axis). Percent single and double positive cells are shown. Gates were set using isotype control antibody for each marker. **(E)** Chip cytometry of BOC-73 cells stained for IGL, κ (red) and λ (green). Inset (lower right), magnified details of two cells, κ and λ positive, respectively. Scale bar, 50μm. **(F)** Flow cytometry of BOC-89 cells stained for IgG1 (x axis) and IgG3 (y axis). Percent single positive and double positive events are shown. Gates were set using isotype control antibodies for each marker. **(G)** Chip cytometry of BOC-89 cells showing IgG1 and IgG3 expression. Inset (lower right), magnified details of marker co-localization (yellow is the result of green and red overlap). Scale bar, 50μm. **(H)** Pie charts showing distribution of the dominant IGH sequence in each of the four cell lines, as determined by BCR ImmunoSeq (productive frequency of top sequence ranging from 93% to 99.9%, as shown, indicating high clonality). Remaining sequences (from top 20) and their productive frequencies are listed in **Supplementary Table 1**. **(I)** Top IGH sequence for each BOC cell line, plotted in panel **(G)**. **(J)**. ImmunoSeq results of BOC-73 cells sorted on IGL,κ and IGL, λ (left and right pie charts, respectively). Parental, unsorted BOC-73 cells are shown in panel **(G)** (arrow). The productive frequency of top, second and third sequences are plotted in orange, yellow and green respectively, and their aa formulas are included in the list below the pie charts. All other sequences (labeled “Remainder”) are collectively shown in dark brown. Top sequence is the same in unsorted BOC-73, and in sorted on kappa or lambda, with frequencies of 93.23%, 99.94%, 48.6% respectively. Complete list of top sequences and their productive frequencies is shown in **Supplementary Table 1**.

To check whether these spontaneously immortalized cell lines, which express CD138 plasma cell marker, are antibody-secreting, we tested the cell culture supernatant for the presence of human immunoglobulins. Two bands, corresponding to immunoglobulin heavy (IGH) and light (IGL) chains were observed in the supernatants of all samples, confirming that all four cell lines are actively secreting antibodies (**Figure 4A**). A single immunoglobulin light chain lambda (IGLλ) was detected in samples BOC-36 (Ab36) and BOC-89 (Ab89), whereas sample BOC-73 (Ab73) revealed two light chain bands. Western blot using IGL-specific antibodies confirmed that BOC-73 sample was positive for both IGL κ and λ (**Figure 4B**).

In line with the flow data in **Figure 1D**, the IGH in sample BOC-78 shows a single, larger band of approximately 75kDa, typical of reduced IgM (51) (**Figure 4A**). Further analyses using isotype detection strips, identified the antibody isotypes secreted by the four cell lines as being IgG1, λ (Ab36), mixture of IgG1,κ and IgG1,λ (Ab73), IgM, κ (Ab78), and a mixture of IgG1,κ and IgG3,κ (Ab89) (**Figure 4C**). The presence of both light chains κ and λ in BOC-73 cells was confirmed by flow cytometry (**Figure 4D**) and chip cytometry (**Figure 4E**), verifying that most cells are light chain κ positive and that κ and λ are separately expressed. FACS analysis of BOC-89 shows prevalence of IgG3, while also showing a dual expressing, IgG1+IgG3+ population. (**Figure 4F**). The presence of double positive cells was also captured via chip cytometry (**Figure 4G**).

BCR sequencing of the variable region (CDR3) of the heavy chain, an approach typically used to determine clonality (52), revealed that all four B lymphoblastoid lines are highly clonal, with one dominant sequence showing productive frequencies of 93.6%, 93.2%, 99.9% and 98.6% in BOC-36, BOC-73, BOC-78, and BOC-89, respectively (**Figure 4H**). The top amino-acid sequence of each BCR is listed in **Figure 4I**, while remainder sequences and their frequencies are listed in **Supplementary Table 1**. In samples BOC-36, BOC-78, and BOC-89, except for the dominant (top listed) sequence, all others occurred with small frequencies (0.5% or less **Supplementary Table 1**), consistent with sequencing artifacts. To further address the clonality of sample BOC-73, which shows mutual expression of immunoglobulin light chain kappa (IGLκ) and lambda (IGLλ), we performed BCR sequencing on the original (mixed, unsorted) population as well as on cells sorted using antibodies specific to either IGLκ or IGLλ. The top sequence (CARGKGQSLGQTSGYKRPFDVW) was enriched from 93.2% in unsorted cells to 99.4% in cells sorted on kappa (**Supplementary Table 1**), revealing that this is the IGH sequence mostly likely pairing with IGLκ. In cells sorted on IGLλ, two other sequences (CARGGSYHSGTGGYYKPPTFDYW and CAREFGTVAWFDPW) showed significant (100 fold) increases in productive frequency, compared to unsorted cells, suggesting that these may represent the two other clonal candidates for lambda (**Figure 4J** and **Supplementary Table 1**). Interestingly, the top sequence in cells sorted on IGLκ (99.4% frequency) is also the top sequence in cells sorted on IGLλ, albeit at a lower (49.6%) productive frequency (**Figure 4J** and **Supplementary Table 1**). This suggests a potential contamination during sorting on IGLλ, with more rapidly proliferating IGLκ positive cells. While co-expression of kappa and lambda light chain in the same cell has been reported before and remains a possibility (**Figure 4D**, half of λ positive cells also co-express κ by flow cytometry), co-localization by fluorescent microscopy could not be observed in these cells (**Figure 4E**, separate expression). Together, the results indicate that BOC-73 (κ) are monoclonal while BOC-73 (λ) may represent a bi-/oligo-clonal population.

### Cell line BOC-36 secretes a monoclonal antibody that recognizes CCDC155

For a detailed analysis of the target antigen, we focused on BOC-36 and the antibody released by this cell line, (Ab36 IgG1,λ **Figures 4A, B**). BCR sequencing of this cell line shows a high frequency of the top sequence (94%, **Figure 4H**), with the rest being distributed among “satellite”, likely artifactual clones, with a low production frequency (0.4% or less, **Supplementary Table 1**). Based on this, we believe BOC-36 cells are very likely monoclonal.

**Figure 5:**
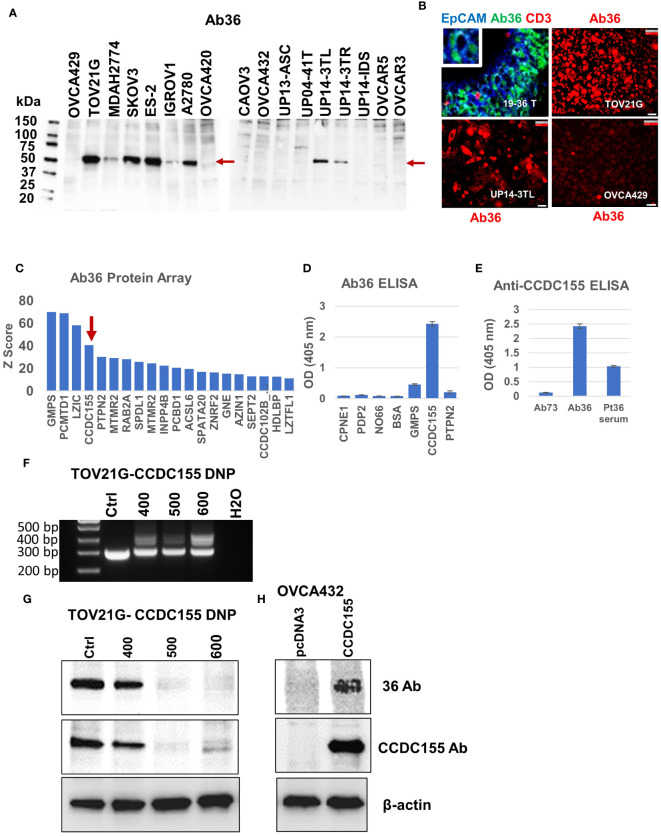
Antibody 36 (Ab36), secreted by BOC-36 recognizes CCDC155 protein. **(A)**. Western blot of cell lysates from 17 different EOC cell lines (listed across), using Ab36. Left lane, MW ladder. Red arrows point to the detected protein, slightly above the 50kDa mark. **(B)** Chip cytometry using Ab36. Upper left panel- staining of a 4 μm section of formalin fixed and paraffin embedded tumor sample 19-36 T, the originating tumor sample for BOC-36, which secretes Ab36. Three antibodies- anti-EpCAM, Ab36 and anti-CD3 are shown in blue green and red, respectively. Upper right, lower left and lower right panels show single marker staining (Ab36, red) in three different EOC cell lines, TOV21G, UP14-3TL and OVCA429, respectively. Scale bar, 50μm. **(C)** Ab36 was tested for binding to more than 21,000 full-length human proteins, using the HuProt™ Array. Secondary antibody binding was quantified using fluorescence intensity measurements. Histogram shows Z score distribution and identity of the top 20 proteins, also listed in **Supplementary Table 2**. Red arrow marks CCDC155. **(D)** ELISA results showing intensity (optical density, OD units) of Ab36 binding to seven different recombinant proteins. Averages of two technical replicates and standard deviations are shown. **(E)** ELISA results using CCDC155 recombinant protein as target. Purified Ab36 and Ab73 (included as control) were used at the same concentration. Samples were run in duplicate, averages and standard deviations are shown. **(F)** CCDC155 was deleted in TOV21G cells, using CRISPR/Cas9 method (CCDC155 double nickase plasmid, DNP). Panel shows PCR products obtained during clone selection at increasing concentrations of selection drug (numbers listed represent puromycin concentration in ng/ml). Cells treated with control DNP were used as reference (Ctrl). **(G)** Western blot using Ab36 probed on cell lysates from parental (Ctrl) cells, or from cells defective in CCDC155, isolated at various concentrations of selection drugs, as described in panel **(F)**. **(H)** Western blot using Ab36 probed on cell lysate from OVCA432 cells, before and after transfection with a CCDC155 encoding plasmid. Beta actin was used as loading control.

Asking if Ab36 can recognize antigens present in EOC tumor cells, we probed Ab36 purified from cell culture supernatant against cell lysates from 17 different human ovarian cancer cell lines. Western blot results show binding in 8 of the 17 human EOC lines, to a target slightly larger than 50kDa (**Figure 5A**). Chip cytometry imaging shows that Ab36 binds to an intracellular protein and confirms that cell lines TOV21G, UP14-TL and OVCA429 express target antigen with high, intermediate, and low expression levels, respectively, in line with Western blot data (**Figure 5B**). When tested against the patient’s own tumor, the staining pattern also showed that Ab36 binds to a target with perinuclear/cytoplasmic distribution. The staining was confined to larger, EpCAM positive, epithelial tumor cells, and was negative in non-tumor cells (such as CD3 T cells, for example) (**Figure 5B**).

For target identification, we queried the purified Ab36 on the HuProt™ human proteome arrays (CDI Labs), comprising more than 21,000 proteins. Intensity of antigen-specific binding was reported using Z score histograms and protein candidates with highest scores were further interrogated. Of the top four proteins (**Figure 5C**), we chose to further test the coiled-coil domain containing 155 protein (CCDC155, also known as KASH5 or Nesprin-5), which has been previously reported to be a high grade serous EOC antigen (53) and which, unlike the other three candidates, has a molecular weight (of 62kDa) that fits the western blot predicted size (**Figure 5A**). Using full length CCDC155 recombinant protein as target (and 6 additional proteins, as controls) we developed an ELISA protocol that confirmed that mAb36 specifically binds to CCDC155 (**Figure 5D**). Importantly, the patient’s serum also showed CCDC155 reactivity, suggesting a systemic humoral response in this patient (**Figure 5E**). In validation of these results, we further demonstrated that binding of Ab36 is lost in TOV21G cells in which we deleted *CCDC155* (**Figures 5F, G**). Additionally, western blot using cell lysate from OVC432 cells (which naturally express undetectable levels) transfected with a plasmid encoding the full length CCDC155 shows increased Ab36 binding, compared to control transfected cells (**Figure 5H**).

**Figure 6:**
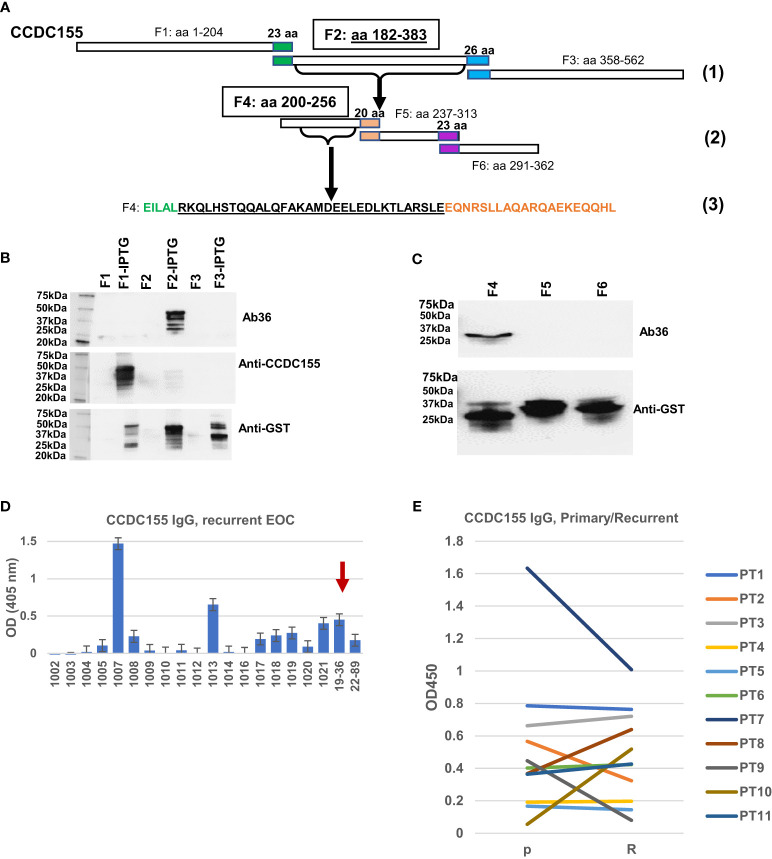
Epitope mapping and CCDC155 immunogenicity. **(A)**. Diagram of the constructs used for epitope mapping, using three steps. Step (1)- whole protein was divided into three, partially overlapping fragments- F1: aa 1 to 204, F2: aa 182 to 383, and F3: aa 358 to 562, respectively. Step (2): F2 was further divided into three partially overlapping fragments (F4, F5 and F6). Step (3): identification of the binding sequence within F4, narrowed down to the 32 amino acids (black font, underlined). **(B)** Western blot of bacterial lysates. *E coli* BL21(DE3) were transformed with vectors containing F1, F2 and F3 (panel A- Step 1, and **Supplementary Figure 3**). Expression was induced with IPTG. Results with and without induction are shown. Cell lysates were loaded onto 4-20% SDS-PAGE gel and transferred onto a nitrocellulose membrane which was stained, in sequence with Ab36 (human IgG1), commercially available, rabbit polyclonal anti-CCDC155 and anti-GST. The membrane was stripped after the first (Ab36) and second (anti-CCDC155) stains. **(C)** Western blot of bacterial lysates. E coli were transformed with vectors containing F4, F5 and F6 (each spanning one third of F2, as described in panel **(A)**- Step 2, and **Supplementary Figure 3**). Results shown are after IPTG induction. Top panel, staining of membrane with Ab36. Bottom panel, membrane staining with anti-GST antibody. **(D)** ELISA results using recurrent EOC patient sera and plates coated with full length recombinant CCDC155 protein. Serum from patient 19-36 (whose tumor constitutes the source of BOC-36) was included as positive control (red arrow). Average of duplicates and standard deviations are shown. **(E)** ELISA measurement of anti-CCDC155 IgG in eleven patients (PT1-PT11) with advanced stage, high grade EOC. Sera from the same patients were collected at the time of diagnosis (primary disease, P) and disease recurrence (R). Average of duplicates are shown.

To map the binding epitope more exactly, we amplified three DNA fragments, each encoding for approximately one third of the full-length CCDC155 protein. Fragment F1 encodes for aa 1 to 204, F2 for aa 182 to 383, and F3 for aa 358 to 562, with a 22-aa overlap between F1 and F2, and a 25-aa overlap between F2 and F3 (**Figure 6A**, step 1). The three PCR fragments were inserted into pGEX4T-1 expression vectors (**Supplementary Figure 3**) and *E coli* BL21(DE3) were transformed with vectors containing F1, F2, F3 fragments, respectively. Bacterial cell lysates show Ab36 binds exclusively to F2 while the commercially available antibody (rabbit polyclonal anti-human CCDC155), included as control, binds to F1 (**Figure 6B**). Fragment 2 was further divided into three smaller domains (F4, F5 and F6, with small overlaps (**Figure 6A** step 2) and Ab36 was found to bind to F4 (**Figure 6C**). This stepwise approach allowed us to narrow down the binding domain to a stretch of 32 amino-acids (**Figure 6A**, step 3- underlined sequence).

To verify the extent to which anti-CCDC155 circulating IgG antibodies are detectable in EOC patient sera, we used a cohort of 20 advanced stage EOC patients, of which 18 had recurrent disease. ELISA results show a detectable response in 9 (45%) of patients (**Figure 6D**). A pairwise comparison in an additional cohort of seven patients with advanced stage, high grade serous EOC shows similar antibody levels in primary and recurrent disease setting (**Figure 6E**), suggesting that patients are largely maintaining the upfront level of antibodies to this antigen. Together, these results further demonstrate that CCDC155 is immunogenic and antigenic in late stage, primary and recurrent EOC, and that the antigen-specific monoclonal antibody Ab36 IgG1, λ, secreted by the spontaneously immortalized BOC-36 cells, captures this phenomenon at a single clone level.

### Antibodies Ab73 and Ab89 recognize PDP2 and GRB2, respectively

**Figure 7:**
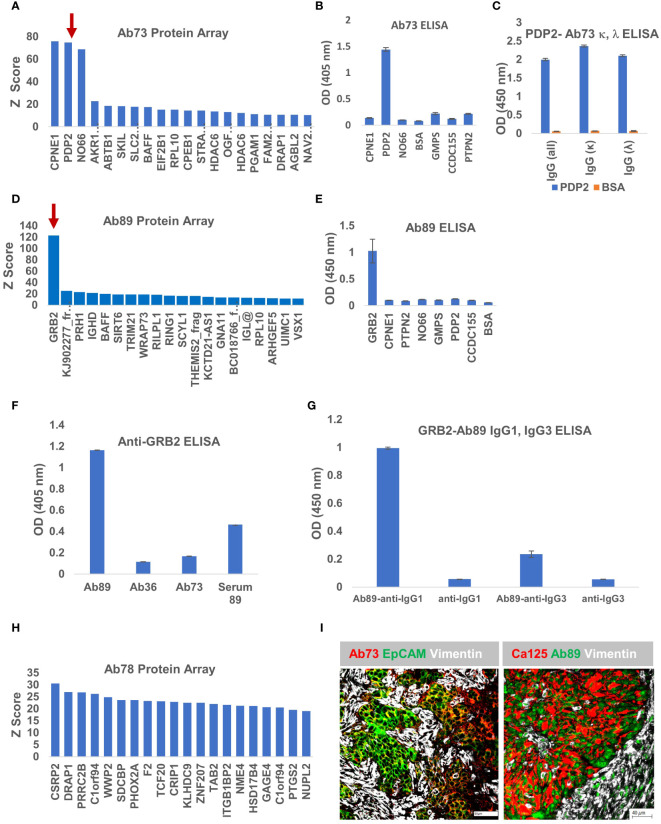
Ab73 and Ab89 target detection using protein arrays. **(A)** Ab73 was tested for binding to more than 21,000 full-length human proteins, using the HuProt™ Array. Secondary antibody binding was quantified using fluorescence intensity measurements. Histogram shows Z score distribution and identity of the top 20 proteins, also listed in **Supplementary Table 2**. Red arrow marks PDP2. **(B)** ELISA results showing intensity (optical density, OD units) of Ab73 binding to seven different recombinant proteins. Averages of two technical replicates and standard deviations are shown. **(C)** ELISA testing of Ab73 binding to PDPD2 recombinant protein (blue bars), or bovine serum albumin (BSA, orange bars) as control. Secondary antibodies to whole IgG, or specific to IgG, kappa or lambda are shown, from left to right, as indicated. Average of duplicate values (OD, 450nm) and standard deviations are shown. **(D)** Ab89 was tested on HuProt™ as described above for Ab36 and Ab73 and as detailed in Materials and Methods. Histogram shows the Z score distribution for the top 20 proteins, listed on the x axis (and in **Supplementary Table 2**). Red arrow marks GRB2. **(E)** ELISA results showing intensity of Ab89 binding to seven different recombinant proteins. Averages of two technical replicates and standard deviations are shown. **(F)** Specific binding to recombinant GRB2 protein was tested by ELISA using purified Ab89 and serum from the same patient (Serum 89). Two controls (Ab36 and Ab73) were used as reference. Anti-human IgG was used as detection antibody. Average of duplicates and standard deviations are shown. **(G)** Binding of Ab89 to GRB2 recombinant protein was tested by ELISA. Secondary antibodies anti-human IgG1 and anti-human IgG3 were used for isotype subclass identification. Average of duplicates and standard deviations are shown. Results from secondary antibodies alone (no primary antibody) serve as background control. **(H)** Z score distribution of Ab78 binding to the HuProt™ array. **(I)** Chip cytometry using Ab73 and Ab89. Four μm section of formalin-fixed and paraffin-embedded tumor samples were used for staining. Left panel - tumor sample 21-73T, the originating tumor sample for BOC-73, which secretes Ab73, was stained with Ab73, anti-EpCAM, and anti-vimentin, shown in red, green, and white, respectively. Scale bar, 50μm. Right panel - tumor sample 22-89T, the originating tumor sample for BOC-89, secreting Ab89, was stained with anti-CA125, Ab89, and anti-vimentin, shown in red, green, and white, respectively. Scale bar, 40μm.

To interrogate the antigenic specificity of the other three clones, we applied the same approach, probing the purified secreted antibody fraction from each cell line, on the HuProt™ human proteome arrays. The Z score distribution of Ab73, purified from BOC-73 unsorted cells, shows three potential targets with high scores: copine 1-CPNE1, pyruvate dehydrogenase phosphatase 2- PDP2, and nucleolar protein NO66 (**Figure 7A**). To further identify which of these candidates is the specific antigen, we developed an ELISA protocol using the three recombinant proteins (and four additional proteins as controls). The results showed that Ab73 (secreted by unsorted cells) specifically binds to PDP2 (**Figure 7B**). Given that the immunoglobulin fraction secreted by unsorted cells comprises two different light chains (IGLκ and IGLλ) (**Figure 4**), we asked which light chain is responsible for binding to PDP2, using IGLκ− and IGLλ - specific secondary antibodies. Interestingly, both IgG1,κ and IgG1,λ bind to PDP2 and the amplitude of the response was equally high, suggesting that the less abundant IgG1,λ may have higher avidity for PDP2 than the prevalent IgG,κ (**Figure 7C**).

Protein array analysis of Ab89 revealed a single candidate -growth factor receptor-bound protein 2, GRB2- as the potential antigenic target (**Figure 7D**). GRB2 is a signaling adapter protein involved in cell signaling pathways, linking receptor tyrosine kinases to MAPK pathways (54). ELISA testing confirmed that Ab89 specifically binds to GRB2 and not to any of the additional 7 proteins, included as non-specific controls (**Figure 7E**). As expected, only Ab89 but not Ab36 or Ab73 (used as negative controls) specifically binds to GRB2 (**Figure 7F**). The serum from the same patient (Serum 89) also shows a positive signal, suggesting a systemic response (**Figure 7F**).

BOC-89 is a highly clonal population, with the top BCR sequence showing a productive frequency of almost 99% (**Figure 4H**). However, two IgG subclasses were detected in BOC-89 supernatant (IgG1 and IgG3) (**Figure 4A**), and some cells seemed to co-express them, with IgG3 being the more abundant fraction (**Figures 4F, G**). To verify which of the two handles binding to GRB2, we used sub-class specific secondary antibodies. The ELISA results revealed that IgG1, the less abundant secreted immunoglobulin, is the one responsible for binding to GRB2, with a much weaker signal coming from IgG3 (**Figure 7G**).

Using a healthy donor PBMC fraction, we similarly generated a fifth B cell line (BHD-1, **Supplementary Figures 4A, B**), which is type I EBV positive (**Supplementary Figures 4C, D**) and is antibody secreting (AbHD1, **Supplementary Figure 4E**). ELISA testing using the purified antibody fraction showed that AbHD1 has no significant binding to CCDC155, PDP2 or GRB2 protein (**Supplementary Figure 4E**), further demonstrating the specific binding of Ab36, Ab78 and Ab89 to their cognate antigens, respectively.

Compared to the other three antibodies described, the Z scores for Ab78 are much lower, and the score distribution shows no clear candidate (**Figure 7H**), raising the possibility that this antibody (of IgM isotype- **Figure 4**), might actually bind to non-peptide epitopes, such as glycans (55).

As with Ab36 (**Figure 5B**), Ab73 and Ab89 also recognize their respective tumor samples (**Figure 7I**). Importantly, their target antigens seem to be expressed by the epithelial tumor cells, highlighted by either EpCAM or CA125 epithelial cell markers, and not by the vimentin-expressing tumor stroma. Together, these results provide a platform for antigenic target identification using tumor-isolated B cells and protein arrays, and reveal CCDC155, PDP2 and GRB2 as the tumor-expressed target antigens recognized by Ab36, Ab73 and Ab89, respectively.

## Discussion

We report here the establishment of 4 novel antibody-secreting B lymphoblastoid cell lines, following spontaneous immortalization of B cells isolated from the tumor microenvironment (tumor tissue or ascites) of high grade serous EOC. The immortalized cells are EBV positive, demonstrating that after the natural viral infection, most likely occurring early in the life of the patient, the latently infected B cells can migrate to the TME. All cells are uniformly positive for the immune memory marker CD27 (56), consistent with EBV latency in the memory B cell compartment. These highly clonal, stably maintained cell lines produce antibodies of either IgG or IgM isotypes. One of the secreted antibodies (Ab36) targets CCDC155, previously reported to be a EOC antigen (53), while two others (Ab73 and Ab89) recognize PDP2 and GRB2, respectively, both with previously unreported antigenic properties in EOC. The repertoire of B cells that infiltrate solid tumor remains largely unexplored (33); the cell lines reported here provide direct evidence of tumor-derived B lymphocyte cell lines that secrete antibodies specific for tumor-expressed targets, and further reinforce the concept that B-TILs, when present, are educated toward tumor-associated antigens (16).

Despite the widely studied associations with cancer pathogenesis, there is a paucity of reports describing the presence of EBV in B TILs, with only scarce evidence showing the presence of EBV-encoded small RNA -positive lymphocytes in gastric adenocarcinoma (57) and a small subset of seminomas (58). To our knowledge, there have been no studies reporting on the EBV status of tumor- or ascites-resident B cells in EOC, a non-EBV related solid tumor type. Our studies demonstrate that oncogenic viral latency could be further exploited for either mechanistic studies on B cell/plasma cell biology following spontaneous immortalization, or for new antigenic target discovery.

While we show the presence of viral EBV proteins after ex vivo culture of B TILs (**Figure 3**), correctly assessing EBV activity inside these cells *in vivo* remains challenging, due to their expected low frequency in the immune TME. It is likely that EBV latency is maintained in B TILs *in vivo*; however, should EBV reactivation occur, this may have potentially important consequences for the antigenic repertoire of the immune TME and the ensuing anti-tumor immune response. Although the exact circumstances are yet to be completely elucidated, it has been shown that EBV reactivation *in vivo* can be experimentally triggered by factors commonly found in the TME, such as TGFβ and hypoxia (59, 60). Additionally, patients’ psychological stress (61, 62) or exposure to certain chemotherapeutics, including cisplatin, commonly used as treatment of EOC, could also cause initiation of viral replication (63, 64).

The quality and quantity of B TILs, and their potential to produce viral antigens should also be considered in the context of lymphocyte/myeloid aggregates and TLS formation. Unlike CD4 and CD8 T cells, which can follow various tumor infiltrating profiles, B TILs are rarely found on their own. Most often, B TILs are found in the proximity of T lymphocytes and other immune cells, contributing to an inflamed, or immunologically ‘hot’ tumor phenotype (11, 17, 46, 47). While the exact spatial location of the isolated EBV+ B cells described here could not be ascertained, all tumor samples were positive for T and B lymphoid aggregates (**Figure 2**). These highly dense lymphocytic infiltrates are engaged in priming and sustaining adaptive immune responses and are often considered to be precursors to TLS. Progression from aggregates to TLS requires stromal rearrangement, presence of certain cytokines or factors such as high antigen load (6, 65). Considering our results, new questions arise on how EBV viral latency and potential viral reactivation in B TILs might contribute to B cell functionality, including viral antigen processing and presentation within the tumor-resident lymphocyte neighborhoods. The hypothesis that immune cell-carrying viral antigens could shape the maturation status of lymphoid aggregates, their stromal rearrangements and potential progression into TLS (66), and consequences on the emerging immune repertoire, is testable and warrants future studies.

Through detailed molecular characterization, we report that Ab36 (IgG1,λ), secreted by monoclonal BOC-36 cells, recognizes CCDC155 (**Figure 5**), a dynein binding protein with roles in connecting the nuclear envelope with the actin cytoskeleton. As part of the nesprin family, CCDC155 is a component of the LINC (LInker of Nucleoskeleton and Cytoskeleton) complex, with important roles in nuclear movement and positioning during telomere attachment to the nuclear envelope in the prophase of meiosis in spermatocytes and oocytes. Outside of a few studies pointing to its roles in male and female gametogenesis (67, 68), CCDC155 biology has been little studied. However, some of the published evidence shows that disruption of the LINC complex triggers defects in cell stability, mobility, and mechano-transduction, all of which could serve as driving forces in tumorigenesis and metastasis (69–74). As part of the nuclear envelope, nesprins bind to lamin A/C, which has been shown to have increased expression in EOC (75). Further studies are needed to identify changes in expression levels and elucidate the inflammatory context in which nesprins (including CCDC155/nesprin5) might become immunogenic. Although no studies have evaluated CCDC155 functionality in ovarian cancer, Wilson et al. reported that circulating anti-CCDC155 IgG levels were significantly elevated in patients with early-stage ovarian cancer compared with healthy controls (53). By finding a tumor-resident B lymphoblastoid cell line with CCDC155 specificity, and by revealing antibodies in almost half of a small cohort of patients (**Figure 6**), our results confirm CCDC155 is a tumor-associated antigen in EOC and point to its continued antigenicity into late-stage, primary and recurrent disease.

We also report that Ab89, secreted by BOC-89 recognizes GRB2 (**Figure 7**), a key adaptor protein that plays a central role in signaling downstream of receptor tyrosine kinases. Signaling via this pathway, which is often dysregulated in EOC, due to ErbB2 and EGFR overexpression or amplification (76), leads to RAS/RAF/mitogen-activated protein kinase (MAPK) and phosphatidylinositol 3-kinase (PI3K)/AKT/mTOR signaling, critically important in tumorigenesis (54, 77). The high prevalence of alterations in these pathways, which are activated in 70% of EOC patients, represents an important therapeutic opportunity. Using liposomal antisense oligodeoxynucleotide, Lara et al. demonstrated that blocking GRB2 expression has therapeutic efficacy in preclinical models of ovarian and uterine cancer (78).

Importantly, we also note that BOC-89 cells seem to co-express IgG1 and IgG3 (**Figure 4**). Challenging the “one B cell-one antibody” rule, single cell sequencing studies have shown that, although most B cells display single V(D)J recombination, two or more V_H_DJ_H_ or V_L_J_L_ recombination patterns can be observed in a single B cell (79). As such, the BOC-89 cell line could offer a versatile tool for gaining insight into multiple Ig expressing, single cell biology.

To date, no studies have reported on spontaneous antibodies to GRB2 in cancer patients. Overexpression of GRB2 can also occur in inflammatory lesions and preneoplastic foci (80), although the exact circumstances have yet to be explored. Similarly, PDP2 reported here as being recognized by Ab73 (**Figure 7**), is an enzymatic modulator of the mitochondrial gatekeeper pyruvate dehydrogenase, with roles in early malignant transformation and a novel therapeutic target (4, 81). There are no reports on PDP2 in ovarian cancer, and the scarce evidence on its roles in controlling metabolism is coming from a limited number of studies, mainly in prostate and lung cancer (82, 83).

Having identified these three self-proteins (CCDC155, GRB2 and PDP2) as antibody targets, our results suggest that, in the right inflammatory context, a break in tolerance occurred. Given the presence of EBV genes in the antibody secreting B cells, one can speculate that this occurred early in life, and that these three proteins could be disease-associated (and later tumor-associated) antigens (84, 85).

Most of the antigens that trigger humoral immunity in cancer patients are intracellular (86, 87). Our findings are in line with this, as CCDC155, GRB2 and PDP2 are all intracellular proteins. Unlike the well-defined immune processes (such as antibody-dependent cellular cytotoxicity-ADCC, and antibody-dependent cellular phagocytosis- ADCP), triggered by antibodies targeting cell surface antigens, the mechanisms by which antibodies to intracellular targets could mediate anti-tumor responses are yet to be fully explored. Evidence shows that antibodies can be internalized by the tumor cells through AP2, important for clathrin-mediated endocytosis (88), and once inside the tumor cell, they can bind to the target via Fab, and to TRIM21 via the Fc receptor (89). Given that TRIM21 is a mediator of ubiquitination which leads to target breakdown (90), the AP2-TRIM21 pathway thereby provides a venue for the intracellular space to be pursued for therapy by antibodies that engage intracellular targets. However, engaging potentially shared antigens in the TME might also heighten the risk of autoimmunity. This possibility is evidenced by the occurrence of paraneoplastic syndromes and immune-related adverse events (irAEs) in patients treated with immune checkpoint inhibitors, where the immune response against the tumor also targets normal tissues (87, 91). The impact of EBV presence in B TILs and its potential reactivation during cancer standard care treatments on these occurrences remains unexplored.

In summary, we reported here that within the TIL population in EOC resides a low frequency population of antibody-secreting B cells that have been naturally exposed to EBV and described a platform for their spontaneous immortalization into highly clonal, B lymphoblastoid cell lines. Once stably maintained, these novel cell lines offer unique opportunities for future studies on B TIL biology and new target antigen recognition, and for studies on EBV latency and/or viral reactivation in the TME of non-EBV solid tumors such as the EOC.

## Data availability statement

The original contributions presented in the study are included in the article/**Supplementary Material**. Further inquiries can be directed to the corresponding authors.

## Ethics statement

The studies involving humans were approved by The Institutional Review Board (IRB) of the University of Pittsburgh. The studies were conducted in accordance with the local legislation and institutional requirements, and written informed consent for participation was obtained.

## Author contributions

LZ: Conceptualization, Data curation, Formal analysis, Investigation, Methodology, Project administration, Resources, Supervision, Validation, Visualization, Writing – original draft, Writing – review & editing. MS: Data curation, Formal analysis, Investigation, Methodology, Project administration, Resources, Validation, Visualization, Writing – original draft, Writing – review & editing. EE: Formal analysis, Investigation, Methodology, Validation, Visualization, Writing – review & editing. SZ: Data curation, Investigation, Methodology, Visualization, Writing – original draft, Writing – review & editing. FM: Data curation, Project administration, Resources, Validation, Visualization, Writing – review & editing. MR: Data curation, Formal analysis, Methodology, Validation, Visualization, Writing – review & editing. RE: Funding acquisition, Investigation, Project administration, Resources, Supervision, Validation, Visualization, Writing – review & editing. OF: Investigation, Methodology, Supervision, Validation, Visualization, Writing – review & editing. AV: Conceptualization, Data curation, Formal analysis, Funding acquisition, Investigation, Methodology, Project administration, Resources, Supervision, Validation, Visualization, Writing – original draft, Writing – review & editing.
