# Supplementary material for: Characterization of latently infected EBV+ antibody-secreting B cells isolated from ovarian tumors and malignant ascites
Source: Front Immunol. 2024 Jul 17;15:1379175. doi: 10.3389/fimmu.2024.1379175 (PMC11288875; doi:10.3389/fimmu.2024.1379175)
Supplement: Supplementary file 1 [file DataSheet_1.pdf]

Suppl Fig. 1

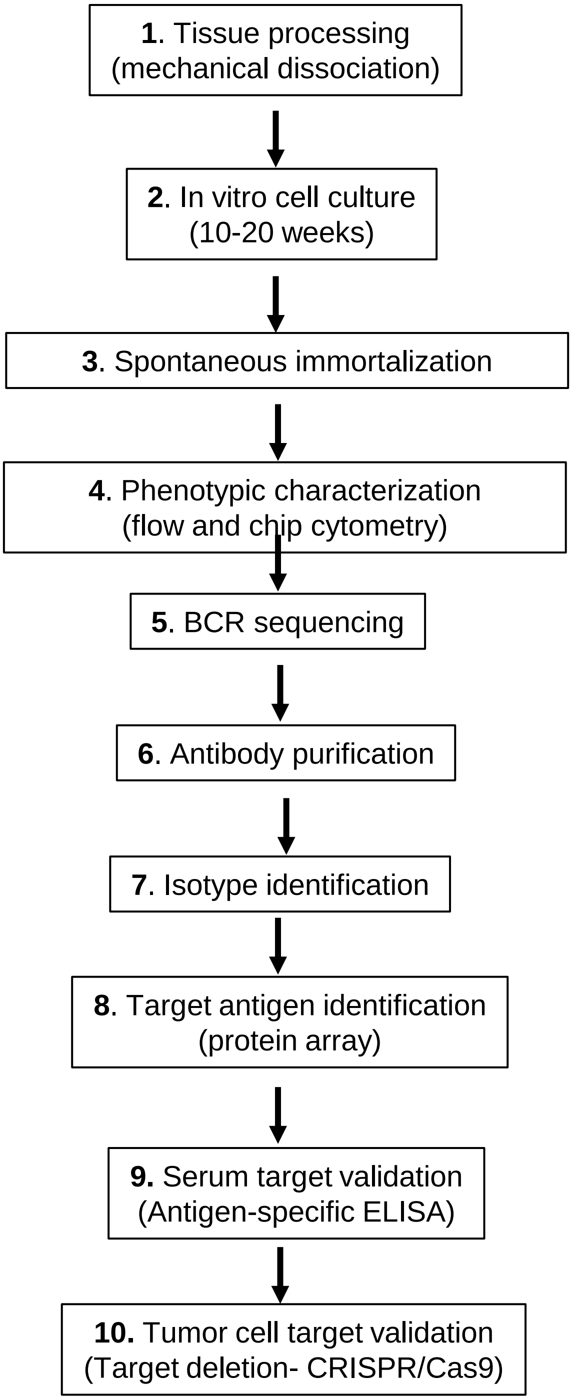

Suppl Fig 1. Experimental design for spontaneous immortalization of EBV+ B TILs, immune phenotyping, antibody isotyping and antigen target identification. All procedures were performed as detailed in Materials and Methods.

Suppl Fig.2

A

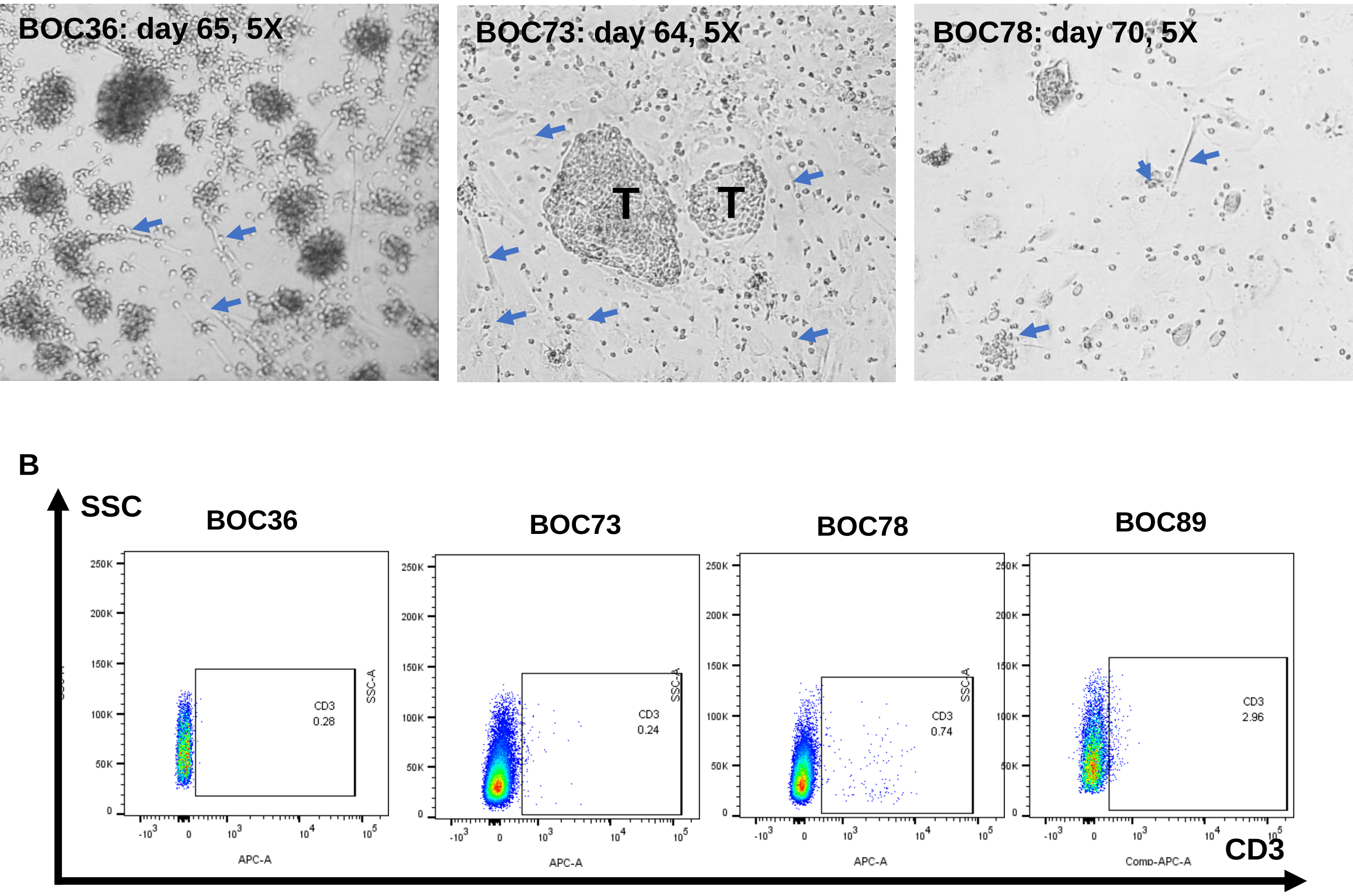

Suppl Fig 2. (A) Cultures of primary cells after in vitro tumor tissue processing shows mixed populations. Following tissue dissociation as described in Suppl Fig 1 and Materials and Methods, cells are propagated in vitro until immortalization is achieved. Images show cellular composition at the indicated numbers of days. The 65-70 days correspond to intermediate time points in culture. T marks tumor cells with epithelial morphology. Blue arrows marks presence of stromal, fibroblast -like cells. The smaller cells scattered throughout in all three samples and organized in rosettes\are lymphoid cells..(B) Flow cytometry staining for CD3 T cell marker. Intensity of labeling of all four cell lines with anti-human CD3-APC labeled antibody is shown on the x axis. Gates were set using isotype control. Y axis –side scatter (SSC)

Suppl Fig 3

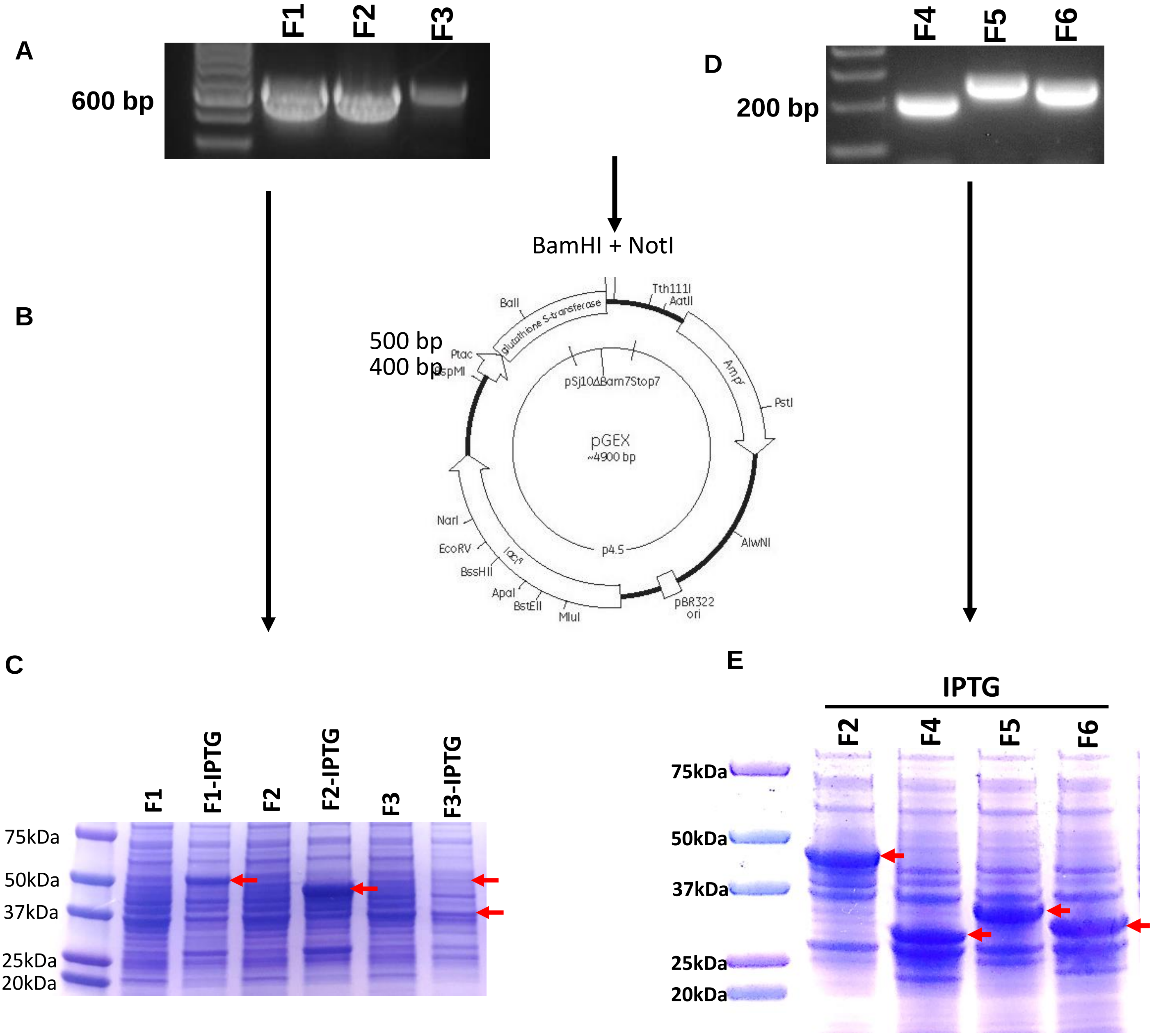

Suppl Fig 3. CCDC155 epitope mapping.(A) Using CCDC155 cDNA as template, three pair of PCR primers were designed to amplify three DNA fragments encoding corresponding protein fragments F1, F2 and F3, shown in Fig 6A (step 1). (B) PCR fragments were inserted into BamHI and NotI digested pGEX4T-1, GST-encoding expression vectors. BL21(DE3) E coli were transformed with vectors containing F1, F2, F3 fragments, respectively. (C) Expression of GST-fusion protein was induced by IPTG (0.5mM) at 37°C for 4 hrs. Cell lysates were loaded onto 4-20% SDS-PAGE gel and transferred onto a NC membrane which was stained, in sequence with Ab36 (human IgG1), anti-CCDC155, and anti-GST (results shown in Fig 6B). Red arrows point to the bands corresponding to the GST-fused protein fragment, expressed after IPTG induction (D) To further narrow down the epitope, F2 was divided into three constructs, F4, F5 and F6, each spanning approximately one third of F2, as shown in Fig 6A (step 2). PCR fragments of F4,5 and 6 are shown and vector expression was performed as described in panel B. (D) IPTG induction of fragments of parental (F2) and three sub-fragments (F4, F5 and F6). Results of SDS-PAGE are shown. All lanes are post-IPTG induction, as indicated. Upon transfer to NC membrane, Western blot was performed, and results are shown in Fig 6C.

Suppl Table 1. Top BCR sequences in four B lymphoblastoid cell lines and their productive frequencies

1A. BCR sequences for cell line BOC-36

**BOC-36**	
Amino Acid	Sum (Productive Frequency)
**CARAGGNYRGFDFW**	**0.935756866**
CATAGGNYRGFDFW	0.004683841
RARAGGTYRGFDFW	0.003885459
CARAGGTYRGFDSW	0.003193528
CARAAANYRGLDFW	0.0029274
CARAGGNYRGFDYW	0.00282095
CARAGAHYRGSDFW	0.00234192
CARAGGNHRGFDFW	0.002235469
CARAGGNYRGFAFW	0.002129019
CASAGRNYRGFDFW	0.002075793
CARAGGHYRAFDLS	0.001969342
CARAGGNYPGFDFW	0.001916117
CARAGGTYRGFDYW	0.001862891
CARAGRNYRGLDFW	0.00175644
CARAGGNYRGLDSW	0.001703215
CARPGGNYRGFDFW	0.001543538
CARAGGYYRGFDFS	0.001383862
CARAGGNYRGFAFS	0.001330637
RARAGGNSPGFDFW	0.001224186
RARAGGNYRGFDFW	0.00117096

1B. ***1A. BCR sequences for cell line BOC-73 (unsorted- top; sorted-bottom)***

BOC-73

BOC-73 Unsorted

Amino Acid	Sum (Productive Frequency)
**CARGKGQSLGQTSGYKRPFDVW**	**0.932344939**
CARGGSYHSGTGGYYKPPTFDYW	0.025516201
CARDFGVVIPFFDYW	0.024439766
CARENYELLTGWEQYWYLDIW	0.00953564
CAREFGTVAWFDPW	0.003890711
CAKDKGGGNIEGVYGMDVW	0.002298057
CAKDQLLMSGGFKYAAVRWDHW	0.000659206
CAALWGNSFPFDYW	0.000556305
CARGSLRGGMDVW	0.000156919
CARHGGYFYSW	0.000149586
CTSSTGHCGGLGCHDAVDVW	9.99686E-05
CARGKGQSLGQTSGYKRPFDDW	9.60579E-05
CARDRDTAMITPGMDVW	7.5282E-05
CAKGGGRSGQSYPFDYW	5.23063E-05
CARENYELLTGWEQYWYLDI	4.47292E-05
CARGKGQSLGQISGYKRPFDVW	2.17536E-05
CARGKGQSLGQTSGYR	1.93093E-05
CARGGSYHSGTGGYYKPPTFDDW	1.19767E-05
CARDFGVVFDDW	1.07546E-05
CARGIGQSLGQTSGYKRPFDVW	8.7992E-06

BOC-73 Sorted

	BOC-73 Sorted on kappa	***BOC-73 Sorted on lambda***
Amino Acid	Sum (Productive Frequency)	Sum (Productive Frequency)
**CARGKGQSLGQTSGYKRPFDVW**	**0.993750045**	**0.496000151**
**CARGGSYHSGTGGYYKPPTFDYW**	0.003576349	**0.338197059**
**CAREFGTVAWFDPW**	0.001009627	**0.136546221**
CARENYELLTGWEQYWYLDIW	0.000675176	0.026599053
CARDFGVVIPFFDYW	0.000730625	0.001744527
CAKDKGGGNIEGVYGMDVW	6.19727E-05	0.000489438
CAALWGNSFPFDYW	8.02885E-05	0.000144804
CARENYELLTGWEQYWYLDI	4.26532E-06	0.000142632
CARGKGQSLGQTSGYKRPFDDW	0.000107135	1.52044E-05

1C. BCR sequences for cell line BOC-78

BOC-78 (kappa)	
Amino Acid	Sum (Productive Frequency)
**CAKDDSGVFDYW**	**0.999437445**
CAKDSSVHTIVVVPAAMPSLVLRYFDWLLSLTTG	0.000357951
CAKDSSVHTIVVVPAA	0.000101672
DSGVFDYW	4.43238E-05
VYYCAKDDSGVFDYW	2.35273E-05
SGVFDYW	2.03763E-05
CAKDSSVHTIVVVPAAMPSLVLRYFDWLLSLTT	5.25163E-06
CAKDSSVHTIVVVPAAMPSLVLRYFDWFIFDYW	3.78118E-06
CAKDSSVHTIVVVPAAMPSLVLRYFDWLLSDYW	2.31072E-06
CAKYDSGVFDYW	2.10065E-06
CAKDSSVHTIVVVPAAMPSLVLRYFDWLLSLTTW	1.26039E-06

1D. BCR sequences for cell line BOC-89

BOC-89	
Amino Acid	Sum (Productive Frequency)
**CAKDHTLSITYVVLFLGPYDSW**	**0.986191028**
CARFNMATIGQSAFDIW	0.009424464
CAREDCSSASCQPDYYYMDVW	0.002034663
CARTRFCSVVSCSYFDRW	0.001033338
CARDHFRGYGGNQNSPAFDIW	0.000362708
CARDHFRGYGGNQNSPVFDIW	0.000109571
CARRGYTNPGPSDCW	8.6634E-05
CAKDHTLSITYVVFFLGPYDSW	6.25415E-05
CAKDHTLSITYVVLFLGPYASC	5.51158E-05
CAKDHTLSITYVVLFLGPSDSW	4.45547E-05
CAKDHSLSITYVVLFLGPYDSW	3.8614E-05
CAKDHTLSITYVVLFLGPHDSW	3.25084E-05
CAKDLSLSITYVVLFLGPYDSW	3.11883E-05
CAREGRGSSDYFDSW	3.03632E-05
CAKDHTLSYVVLFLGPYDSW	2.44226E-05
CAKDHTLSITYVVLLLGPYDSR	2.24423E-05
CAKDHTLSITYVVPFLGPYDPR	2.21123E-05
CAKDQTLSITYVVLFLGPYDSW	2.17823E-05
CAKDHTLSITYVLFLGPYDSW	2.16173E-05
CAKDHTLSITYVVLVVLFLGPYDSW	2.11222E-05

Suppl Table 2. Z score distribution for top 20 target candidates, as determined by the human protein array HuProt™

Ab36		Ab73		Ab78		Ab89	
**Name**	**Z Score**	**Name**	**Z Score**	**Name**	**Z Score**	**Name**	**Z Score**
GMPS	70.12	CPNE1	75.91	CSRP2	30.556	GRB2	123.138
PCMTD1	68.971	PDP2	74.944	DRAP1	27.025	KJ902277_frag	25.269
LZIC	58.331	NO66	68.788	PRRC2B	26.875	PRH1	23.21
CCDC155	40.847	AKR1B1	22.697	C1orf94	26.229	IGHD	21.768
PTPN2	30.31	ABTB1	18.488	WWP2	24.894	BAFF	19.756
MTMR2	29.116	SKIL	18.378	SDCBP	23.603	SIRT6	19.011
RAB2A	28.211	SLC27A3	17.813	PHOX2A	23.581	TRIM21	18.744
SPDL1	25.935	BAFF	17.499	F2	23.215	WRAP73	18.686
MTMR2	24.412	EIF2B1	15.151	TCF20	23.086	RILPL1	18.162
INPP4B	22.521	RPL10	15.104	CRIP1	22.914	RING1	17.069
PCBD1	20.554	CPEB1	14.436	KLHDC9	22.548	SCYL1	16.173
ACSL6	19.517	STRA13	14.256	ZNF207	22.527	THEMIS2_frag	16.103
SPATA20	17.031	HDAC6	13.604	TAB2	22.032	KCTD21-AS1	14.836
ZNRF2	16.368	OGFOD3	13.188	ITGB1BP2	21.644	GNA11	13.859
GNE	15.413	HDAC6	12.269	NME4	21.3	BC018766_frag	13.428
AZIN1	14.81	PGAM1	11.217	HSD17B4	21.214	IGL@	13.405
SEPT2	13.161	FAM21A	10.762	GAGE4	20.611	RPL10	12.777
CCDC102B_frag	13.135	DRAP1	10.675	C1orf94	20.546	ARHGEF5	12.137
HDLBP	12.751	AGBL2	10.62	PTGS2	19.556	UIMC1	11.835
LZTFL1	11.097	NAV2_frag	10.424	NUPL2	19.083	VSX1	11.555

Suppl Fig. 4

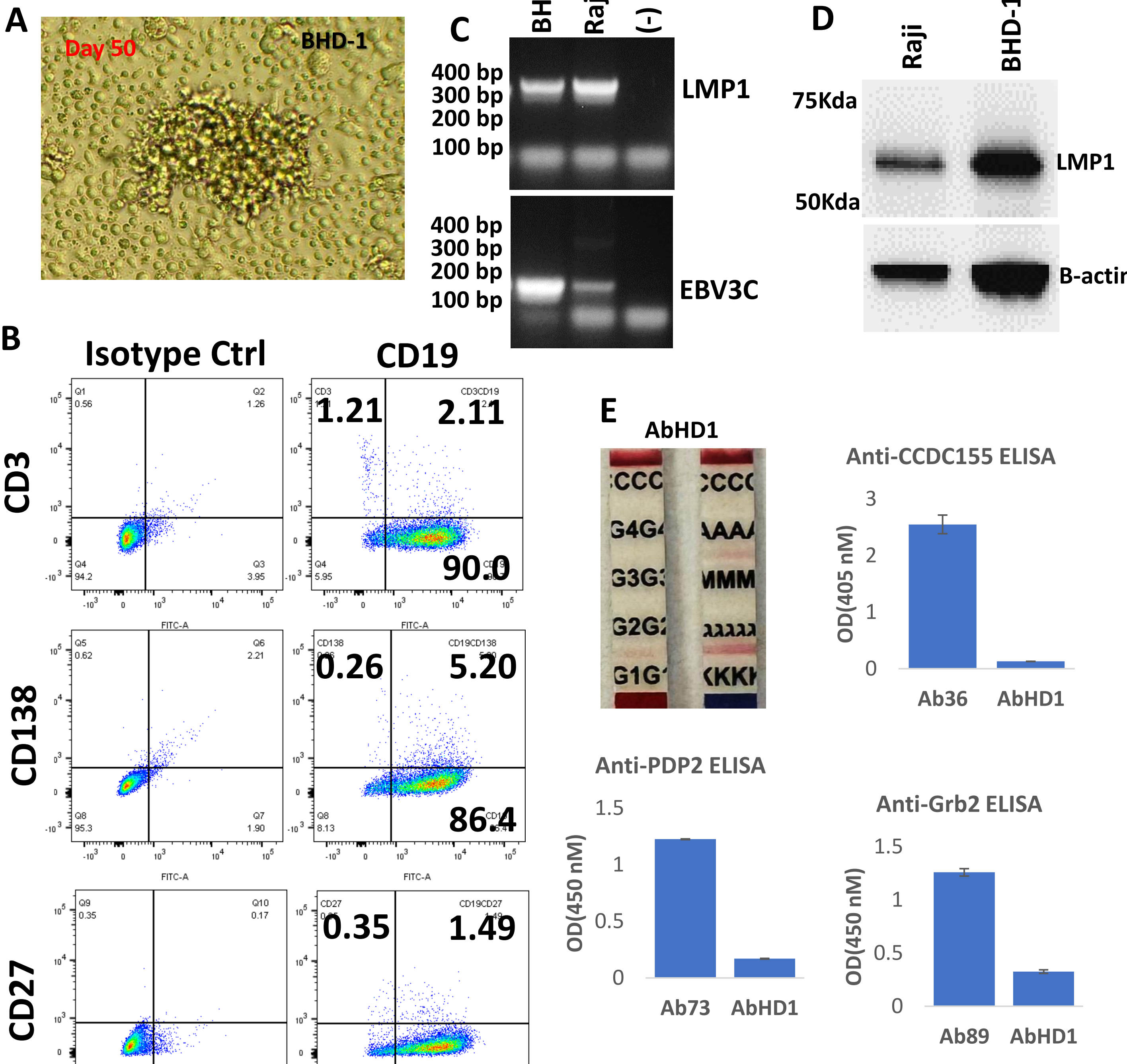

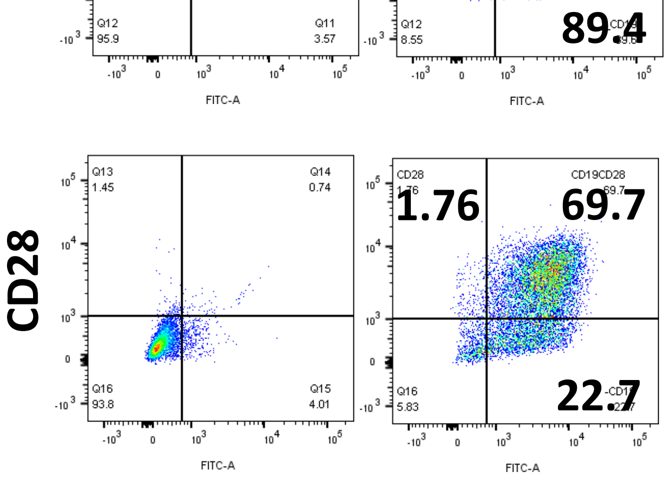

Suppl Fig.4. Immortalized B cells from PBMCs of a healthy donor (BHD-1) are EBV positive and secrete AbHD1. A. Cell clusters after 50 days in culture. B. Flow cytometry analysis of B cell markers. C. Genomic PCR showed that the BHD-1 cells are type I EBV positive . D. Western blot results showed the expression of EBV LMP1 expression in the BHD-1 cells with Raji cells as positive control. E. Antibodies (AbHD1) generated by the BHD-1 cells are a combination of IgG1 and IgM with kappa light chains. ELISA results using Ab36, Ab73 and Ab89 as positive controls, respectively.
